# ERRα fosters running endurance by driving myofiber aerobic transformation and fuel efficiency

**DOI:** 10.1016/j.molmet.2023.101814

**Published:** 2023-10-05

**Authors:** Hui Xia, Charlotte Scholtes, Catherine R. Dufour, Christina Guluzian, Vincent Giguère

**Affiliations:** 1 Goodman Cancer Institute, McGill University, Montréal, Québec, Canada H3A 1A3; 2 Department of Biochemistry, Faculty of Medicine and Health Sciences, McGill University, Montréal, Québec, Canada H3G 1Y6

**Keywords:** Diabetes, Endurance exercise, Fuel metabolism, Lactate, Mitochondrial oxidative metabolism, Muscle function, Muscle wasting, Myofibers, Nuclear receptor

## Abstract

**Objective:**

Estrogen related receptor α (ERRα) occupies a central node in the transcriptional control of energy metabolism, including in skeletal muscle, but whether modulation of its activity can directly contribute to extend endurance to exercise remains to be investigated. The goal of this study was to characterize the benefit of mice engineered to express a physiologically relevant activated form of ERRα on skeletal muscle exercise metabolism and performance.

**Methods:**

We recently shown that mutational inactivation of three regulated phosphosites in the amino terminal domain of the nuclear receptor ERRα impedes its degradation, leading to an accumulation of ERRα proteins and perturbation of metabolic homeostasis in ERRα^3SA^ mutant mice. Herein, we used a multi-omics approach in combination with physical endurance tests to ascertain the consequences of expressing the constitutively active phospho-deficient ERRα^3SA^ form on muscle exercise performance and energy metabolism.

**Results:**

Genetic heightening of ERRα activity enhanced exercise capacity, fatigue-resistance, and endurance. This phenotype resulted from extensive reprogramming of ERRα global DNA occupancy and transcriptome in muscle leading to an increase in oxidative fibers, mitochondrial biogenesis, fatty acid oxidation, and lactate homeostasis.

**Conclusion:**

Our findings support the potential to enhance physical performance and exercise-induced health benefits by targeting molecular pathways regulating ERRα transcriptional activity.

## Highlights

• A physiologically relevant phospho-deficient ERRα^3SA^ mouse model displays improved exercise performance.
• ERRα^3SA^ mice exhibit a reprogrammed skeletal muscle ERRα cistrome.
• ERRα^3SA^ drives myofiber aerobic transformation and metabolic fuel efficiency.
• ERRα^3SA^ enhances lactate clearance by augmenting its oxidation to pyruvate.

## Abbreviations

AICAR: 5-aminoimidazole-4-carboxamide ribonucleotide
AMPK: AMP kinase
ANT: adenine nucleotide translocator
ChIP: chromatin immunoprecipitation
CoA: coenzyme A
DEGs: differentially expressed genes
ERR: estrogen-related receptor
ERRE: ERR response element
eWAT: epididymal white adipose tissue
FA: fatty acid
FAO: fatty acid oxidation
FFA: free fatty acid
LD: lipid droplet
LDH: lactate dehydrogenase
LTT: lactate tolerance test
mtDNA: mitochondrial DNA
NCoR1: nuclear receptor corepressor 1
OxPhos: oxidative phosphorylation
PCR: polymerase chain reaction
PGC-1: peroxisome proliferator-activated receptor γ coactivator 1
RIP140: receptor interactor protein 140
ROS: reactive oxygen species
SDH: succinate dehydrogenase
TCA: tricarboxylic acid
WT: wild type

## 1 Introduction

Physical exercise elicits a multitude of benefits, mainly reflected by body weight loss, glucose homeostasis, insulin sensitivity, mitochondrial content/function, metabolic flexibility, as well as effects on the cardiovascular system, longevity, and tumor control [[1], [2], [3], [4], [5], [6]]. Thus, identifying agents that would mimic or potentiate the genetic effects of exercise to treat the related metabolic diseases is of great interest [7,8]. Despite progress in the field, the longstanding goal to identify targetable signaling pathways and transcription factors to improve endurance and muscle functions is still elusive.

Skeletal muscle is highly plastic even when nutrient availability is suboptimal. Muscle efficiently utilizes diverse energy sources (e.g., carbohydrates, fatty acids, amino acids, ketone bodies) and displays great flexibility in metabolic fuel selection [[9], [10], [11]]. This capacity relies greatly on the heterogeneity of muscle fibers, which are commonly classified into two types based on contraction speed, fatigability, and energy metabolism. The slow-twitch type I fibers have high mitochondrial content and preferentially use oxidative metabolism. Type II fibers exhibit faster twitch attributes and are subdivided into three categories. Type IIB have low mitochondrial levels and are specialized for anaerobic glycolytic metabolism. Type IIA possess more mitochondria than type IIB but are more glycolytic compared to type I, thus are mixed oxidative-glycolytic. Type IIX fibers are intermediate between IIA and IIB in metabolic and contractile properties and are often more oxidative and fatigue-resistant than IIB fibers [12].

Transcriptional regulation rigorously couples environmental demands with muscle distinctions, such as fiber size, metabolism, and contractile modes. Nuclear receptor ERRα and its co-regulators PGC-1α/β, RIP140 and NCoR1 have been established to feed into a highly complex transcriptional network that act in concert to control energy metabolism and muscle functions [[13], [14], [15], [16], [17], [18], [19], [20]]. ERRα expression has been reported to be transcriptionally induced by exercise in skeletal muscle of both mice and humans [[21], [22], [23]]. ERRα-null mice display exercise intolerance, an effect found exacerbated by the combined loss-of-function of ERRα and ERRγ in muscle [14,24,25]. ERRα targets are also downregulated in muscles of patients with metabolic abnormalities [26,27]. We recently demonstrated that the activity of ERRα is strictly regulated by phosphorylation in response to insulin signaling, leading to changes in its cellular localization, ubiquitylation, and protein stability [28]. Mice engineered to express an ERRα protein harboring mutations in three phosphosites within the amino terminal domain of ERRα (ERRα^3SA^) display lasting ERRα protein stabilization accompanied by reduced insulin sensitivity despite improved mitochondrial function. These observations prompted us to explore the impact of augmented ERRα activity on exercise metabolism and muscle performance.

In this study, we demonstrate that ERRα^3SA^ phospho-mutant mice exhibit increased exercise endurance associated with improved lactate homeostasis, enhanced muscle mitochondrial function and oxidative capacity. This phenotype arises from an increase in ERRα occupancy and binding efficacy at specific sites on chromatin, leading to the activation of a genetic program mimicking physiological impacts of exercise training. Our results suggest that targeting an ERRα activating pathway might be used to improve exercise capacity, muscle functions, and the management of related metabolic diseases.

## 2 Materials and methods

### 2.1 Cell culture

C2C12 cells were obtained from the ATCC and cultured in DMEM (cat. no. 11965092; Thermo Fisher Scientific) supplemented with 10 % (v/v) fetal bovine serum (FBS; cat. no. 12483020; Thermo Fisher Scientific), 100 units/ml penicillin-streptomycin (Wisent), and 1X sodium pyruvate (Wisent) at 37 °C in a humidified incubator containing 5 % CO_2_. Cells were periodically tested for mycoplasma contamination using a mycoplasma PCR detection kit (cat. no. G238; Applied Biological Materials) and showed no signs of infection. C2C12 cells were treated with 1 mM AICAR for 6 h (cat. no. A9978; Sigma–Aldrich) as described in the corresponding legend.

### 2.2 Mice

All mouse manipulations were performed in accordance with procedures approved by the McGill Facility Animal Care Committee within animal protocol 3173 and complied with ethical guidelines set by the Canadian Council of Animal Care. Unless otherwise specified, all experiments used age-matched male littermates (2- to 3-month-old). Mice were housed two to five per cage in a constant environment (ambient temperature: 22°C–24 °C; relative humidity: 30%–70 %) under a 12-h light/dark cycle (7 am-7 pm light, 7 pm-7 am dark) with ad libitum access to water and a standard normal diet (ND; Envigo; Teklad Rodent diet 2920×; 3.1 kal/g, 24 kcal% protein, 16 kcal% fat, 60 kcal% carbohydrate) in an animal facility at McGill University. Mice were euthanized by cervical dislocation for serum and tissue isolations. Skeletal muscle (gastrocnemius and soleus), quadriceps, liver, heart, eWAT were harvested and snap-frozen in liquid nitrogen and stored at −80 °C until processing. ERRα^3SA^ phospho-mutant mice on a C57BL/6 N genetic background have been described previously [28].

### 2.3 Preparation of cell or tissue lysates and immunoblotting

For analyzing whole cell extracts of cultured C2C12 cells, cells were washed in ice-cold PBS and lysed using buffer K consisting of 20 mM Phosphate Buffer pH 7.0, 15 mM NaCl, 1 % NP40, 5 mM EDTA, as well as protease and phosphatase inhibitors (Thermo Fisher Scientific). For tissue lysates, frozen mouse tissues were pulverized in liquid nitrogen followed by homogenization and sonication in buffer K.

Protein lysates were subjected to determination of concentration using Bio-Rad protein assay dye reagent (cat. no. 5000006). After boiling with SDS sample buffer, denatured proteins were separated by SDS-PAGE, transferred to PVDF membranes (Bio-Rad), blotted according to primary antibody manufacturers' recommendations, and detected using ECL select (cat. no. CA97068-824; Amersham Biosciences), Clarity ECL (cat. no. 1705061; Bio-Rad) or Clarity Max ECL (cat. no. 1705062; Bio-Rad) Western blotting detection reagent. Data were collected using a ChemiDoc MP imaging System (Bio-Rad). The following antibodies were used: ERRα (1:1000 dilution; cat. no. ab76228; Abcam); phospho-ERRα (Ser19) (1:1000 dilution, homemade [29]; Phospho-AMPKα (Thr172) (1:1000 dilution; cat. no. 2535S; Cell Signaling Technology); AMPKα (1:1000 dilution; cat. no. 2532S; Cell Signaling Technology); Vinculin (1:2000 dilution; cat. no. MAB3574; clone VIIF9; Sigma–Aldrich); Tubulin (1:5000 dilution; cat. no. CLT9002; clone DM1A; Cedarlane); PGC1α (1:200 dilution; cat. no. sc-13067; Santa Cruz Biotechnology); LDHA (1:1000 dilution; cat. no. 2012S; Cell Signaling Technology); LDHB (1:1000 dilution; cat. no. A7625; ABclonal); MCT1 (1:1000 dilution; cat. no. A3013; ABclonal); MCT4 (1:1000 dilution; cat. no. A10548; ABclonal); total OxPhos rodent antibody cocktail (1:2000 dilution; cat. no. ab110413 (MS604); Abcam); MyHC I (1:2000 dilution; cat. no. BA-D5; DSHB); MyHC IIA (1:2000 dilution; cat. no. SC-71; DSHB); MyHC IIB (1:2000 dilution; cat. no. BF-F3; DSHB); Myoglobin (1:1000 dilution; cat. no. A5471; ABclonal); VEGFA (1:1000 dilution; cat. no. A5708; ABclonal); FABP3 (1:1000 dilution; cat. no. A5312; ABclonal); ACADL (1:2000 dilution; cat. no. A1266; ABclonal); PDK4 (1:1000 dilution; cat. no. A13337; ABclonal); phospho-HSL (Ser563) (1:1000 dilution; cat. no. 4139; Cell Signaling Technology); phospho-HSL (Ser660) (1:1000 dilution; cat. no. 4126; Cell Signaling Technology); HSL (1:1000 dilution; cat. no. 41078; Cell Signaling Technology); ATGL (1:1000 dilution; cat. no. A6245; ABclonal); Perilipin-1 (1:1000 dilution; cat. no. 9349; Cell Signaling Technology); NDUFB3 (1:1000 dilution; cat. no. A14378; ABclonal); SDHB (1:5000 dilution; cat. no. ab14714; Abcam); UQCRQ (1:1000 dilution; cat. no. A9872; ABclonal); COX7A2 (1:1000 dilution; cat. no. A8406; ABclonal); ATP5K (1:1000 dilution; cat. no. A16769; ABclonal); Cytochrome c (1:1000 dilution; cat. no. A13430; ABclonal).

### 2.4 RNA extraction and quantitative real-time PCR (RT-qPCR)

Total RNA of C2C12 cells, liver and heart was extracted using a RNeasy Mini Kit (cat. no. 74106; QIAGEN). Muscle RNA was extracted using a RNeasy Fibrous Tissue Mini Kit (cat. no. 74704; QIAGEN). RNA of adipose tissue was extracted using a RNeasy Lipid Tissue Mini Kit (cat. no. 74804; QIAGEN). Isolated RNA was then reverse-transcribed using ProtoScript II Reverse Transcriptase (cat. no. M0368X; NEB) according to the manufacturer's instructions. Synthesized cDNA was amplified by RT-qPCR with SYBR Green Master Mix (cat. no. 4887352001; Roche) on a LightCycler 480 instrument (Roche). The relative expression levels of selected mouse genes were normalized to the housekeeping gene *B-actin*, *Hprt*, or *Arbp*. Specific primer sequences are listed in Suppl. Table 3.

### 2.5 ChIP-sequencing

ERRα ChIP-seq experiments were performed using liver or skeletal muscle (gastrocnemius and soleus) of wild-type (WT) and ERRα^3SA^ mice sacrificed at zeitgeber time 9 by cervical dislocation.

For liver ChIPs, livers of two mice per genotype were pooled and homogenized with a polytron (power 4) in 10 ml of ice cold 1X PBS and then centrifuged at 3000 rpm for 2 min at 4 °C. Supernatants were removed and pellets were resuspended in 10 ml RT 1X PBS containing 1 % formaldehyde (cat. no. 2106-01; JT Baker) and protease inhibitors and left to rotate at RT for 12 min. Tubes were kept on ice and centrifuged at 3000 rpm for 2 min at 4 °C followed by three washes with 10 ml of ice-cold 1X PBS. Then, cell pellets were resuspended with 10 ml of cell lysis buffer (5 mM HEPES pH 8, 85 mM KCl, 0.5 % NP-40 and protease inhibitors). After a quick homogenization with a polytron (power 4), tubes were incubated at 4 °C for 30 min with rotation and vortexed every 10 min. Centrifugation was carried out at 3000 rpm for 10 min at 4 °C and supernatants were removed to obtain nuclear pellets. Nuclear pellets were resuspended in 4 ml of sonication buffer per genotype (50 mM Tris–HCl pH 8.1, 10 mM EDTA, 1 % SDS and protease inhibitors), transferred to Eppendorf tubes in 500 μl aliquots and sonicated (Fisher Scientific model 100) at power 10 for 8 s pulses 40 times in a water/ice bath in a cold room. Samples were subjected to centrifugation at 13,000 rpm for 10 min at 4 °C and supernatants were collected in new tubes. To test the efficacy of chromatin sonication, 20 μl of sonicated chromatin was transferred into a tube with 200 μl of decrosslinking buffer (1 % SDS, 0.1 M NaHCO_3_), 8 μl of NaCl (5 M) and 10 μl of 1 mg/ml proteinase K (cat. no. PRK403.250; BioShop). Tubes were incubated at 65 °C for 1 h for a fast decrosslink. Samples were purified using a Qiaquick PCR purification kit (cat. no. 28106; Qiagen) and eluted with 30 μl of homemade elution buffer (10 mM Tris–HCl pH 8.0, 0.1 mM EDTA pH 8.0). The samples were run on a 1 % agarose gel with ethidium bromide to ensure the observation of sheared chromatin between 200 and 1000 bp with a concentration around 500 bp. One day prior, 60 μl of magnetic Protein G Dynabeads were washed twice with 1 ml of cold blocking buffer (1X PBS, 0.5 % BSA) and incubated overnight in 400 μl of cold blocking solution with 10 μl of anti-ERRα antibody (cat. no. ab76228; Abcam) at 4 °C with rotation. Then, supernatants were discarded, and beads were washed twice with 1 ml of cold blocking solution. Subsequently, 200 μg of chromatin diluted in 2.5X ChIP dilution buffer (2 mM EDTA pH 8.0, 100 mM NaCl, 20 mM Tris–HCl pH 8.0, 0.5% Triton X-100, and protease inhibitors) and 100 μl blocking buffer were added to the antibody-bound beads and kept for overnight rotation at 4 °C. The next day, beads were washed 6 times for 3 min each at 4 °C with rotation using 1 ml of LiCl buffer (100 mM Tris–HCl pH 7.5, 500 mM LiCl, 1 % NP-40 and 1 % Na-desoxycholate). For the fourth wash, beads were transferred to a new tube. Then, beads were washed quickly once with 1 ml of ice-cold TE buffer (10 mM Tris–HCl pH 8.0 and 1 mM EDTA pH 8.0). 300 μl of decrosslink buffer was added followed by vortex and overnight incubation in a 65 °C water bath. The following day, tubes were centrifuged at 13,000 rpm for 3 min at RT. Supernatants were collected in a new tube and 300 μl of TE buffer was added. 1.2 μl of RNase A (cat. no. 79254; Qiagen, 0.2 μg/μl final) was added to 600 μl of sample and tubes were kept at 37 °C for 2 h. Then, 12 μl proteinase K (0.2 μg/μl final) was added and incubated at 55 °C for 2 h. Chromatin from four ChIPs per genotype were purified using a QIAquick PCR purification kit (cat. no. 28106; Qiagen) and eluted using the same 37 μl of homemade elution buffer (10 mM Tris–HCl pH 8.0, 0.1 mM EDTA pH 8.0).

For skeletal muscle ChIPs, muscles from both hind limbs of five mice per genotype were pooled and homogenized with a polytron (power 5) in 10 ml of ice cold 1X PBS and then centrifuged at 3000 rpm for 2 min at 4 °C. Muscle ChIP DNA were obtained as described above for liver ChIPs with the following discrepancies. Nuclear isolation was performed prior to the crosslinking step. For sonication, crosslinked nuclei were resuspended in 3 ml of sonication buffer per genotype, transferred to Eppendorf tubes in 500 μl aliquots and sonicated at power 10 for 8 s pulses 60 times in a water/ice bath in a cold room. 170 μg of chromatin was added to 60 μl antibody-bound beads linked with 10 μl of ERRα antibody and kept for overnight rotation at 4 °C.

Liver and skeletal muscle ChIP DNA prepared as described above were provided to the Génome Québec Innovation Centre for DNA library preparation using the NEB Ultra II DNA library preparation kit according to Illumina recommendations. ChIP DNA libraries were sequenced using the NovaSeq 6000 platform (Illumina) as 100bp paired-end reads. ChIP-seq reads were first trimmed for adapter sequences and low-quality score bases using Trimmomatic v0.36 [30]. The resulting reads were mapped to the mouse reference genome (mm10) using BWA-MEM v0.7.12 [31] in paired-end mode at default parameters. Only reads that had a unique alignment (mapping quality >20) were retained and PCR duplicates were removed using Picard tools v2.0.1 (https://broadinstitute.github.io/picard/). Peaks (summit +/− 150 bp) were called using MACS2 software suite v2.1.1.20160309 [32] with the default FDR (alpha = 0.05) and further filtered to FDR <0.001 using sequenced libraries of input DNA as control. Peaks in mitochondrial chromosome and scaffold regions were removed. Peak annotation and *de novo* motif enrichment analyses were performed using the annotatePeaks and findMotifsGenome commands, respectively, from HOMER software suite v4.9.1 [33]. Peak intersection analysis was performed with HOMER using the script *mergePeaks -d300* on the total list of peaks with subsequent filtering for peaks annotated to genes ±20 kb of TSS to determine peaks specific to WT, ERRα^3SA^ or common to both. Separate “reference peak sets” was generated by merging ChIP-seq peaks across samples in the same experiment, using bedtools merge v2.27.0 with parameters: -sorted -d −150 (https://bedtools.readthedocs.io/). Peak signals were then calculated as Fragments Per Kilobase of transcript per Million mapped reads (FPKM) using HOMER. ERRα ChIP-seq tracks were visualized using IGV (v2.11.2) [34].

### 2.6 Treadmill exhaustion test

Age-matched (3-month-old) ERRα^3SA^ mice or control littermates were exercised on a five-lane rodent treadmill (Harvard Apparatus). Mice were pre-adapted to the treadmill for 3 days with the following program. Day 1 - static treadmill band 10 min. Day 2 - walking on the treadmill for 10 min (8.3 cm/s). Day 3 -running for 10 min (16.6 cm/s). Electric stimulus of 0.3 mA was employed to force mice to run. The run-to-exhaustion test was conducted by gradually increasing speed to 35 cm/s in 24 min followed by the exhaustion run at 35 cm/s until mice failed. Exhaustion was considered after 5 s permanence on the electric grid. If the mouse remained on the treadmill for more than 120 min, mice were removed from the machine and the test was considered as completed. Maximum exercise capacity was estimated from each run-to-exhaustion trial using three parameters: the duration of the run (min), the distance ran (m), and the work performed (J), with work being the product of body weight (kilograms), gravity (9.81 m/s^2^), vertical speed (meters per second times angle), and time (seconds). Mice were put back into their respective cages with free access to water and food and sacrificed 3.5 h post endurance running. A second experiment was carried out to confirm that the upregulation of ERRα protein levels observed in response to the exercise regimen was not influenced by potential post-feeding insulin-mediated induction of ERRα. In the second experiment, exercised mice did not have access to food (only water) during the recovery period (3.5 h) and the control sedentary group had no access to food from the start of the exercise test until the end of the recovery period of the exercised group.

### 2.7 Rotarod test

Motor coordination ability was examined by two tests using a five-lane rotarod (IITC Life Science). For the first test, the rotarod was set to accelerate steadily from 4 to 40 rpm for 300 s, mice (3.5-month-old) were habituated to the rotarod for 5 min on the first day and then the animals were subjected to three trials per day (with 15 min inter-trial rest) for five consecutive days. The second test was performed on the seventh day at constant speed (20 rpm for 600 s). Mice remaining on the rod for more than 10 min were removed from the machine and the test was considered as completed. The duration that each animal was able to stay on the rotating rod was recorded as the time to fall and only the average score among the three trials was considered for analysis.

### 2.8 Grip strength test

Maximum muscle strength of the 3.5-month-old ERRα^3SA^ mice and their age-matched WT littermates was measured using a grip strength meter (Columbus instruments). The mouse was allowed to grab the metal grid with four limbs and gently pulled backwards in a horizontal line by the tail until the grip was released. The peak force applied to the grip by the animal at the time of the release is recorded as maximum strength (Newtons). Three measurements were collected from each animal for 3 consecutive days and the average value was used for statistical analysis.

### 2.9 Biochemistry measurements

Mouse blood samples were collected from the submandibular vein of mice. Blood glucose and lactate were measured from tail lateral vein blood using a OneTouch Ultra®2 glucose meter (LifeScan) and Lactate Scout (Lactate.com) in 4-month-old WT and ERRα^3SA^ mic before exercise, upon exhaustion of the first mouse and at failure in the treadmill endurance test, respectively. Muscle FFA and glycogen contents were determined using a Free Fatty Acid Assay Kit (cat. no. ab65341; Abcam) and a Glycogen Assay Kit (cat. no. ab65620; Abcam) respectively, as per the manufacturer's recommendations.

### 2.10 Citrate synthase assay

Citrate synthase (CS) activity was measured using a Citrate Synthase Assay Kit (cat. no. ab239712; Abcam). About 10 mg frozen quadriceps of WT and ERRα^3SA^ mice were homogenized with ice-cold CS Assay Buffer (200 μl per 10 mg tissue), quickly sonicated, and left on ice for 10 min followed by centrifugation at 11,000 rpm for 5 min. Briefly, 50 μl supernatants of each sample were added in a 96-well plate in duplicate, mixed with either 50 μl control solution or 50 μl reaction solution, and the colorimetric reaction was read immediately and every 5 min in kinetic mode for 60 min at 405 nm on the Azure Ao absorbance microplate reader.

### 2.11 Fatty acid oxidation assay

FAO activity was measured using a Fatty Acid Oxidation Kit (cat. no. E-141; Biomedical Research Service). The method is based on oxidation of the substrate octanoyl-CoA coupled to FADH_2_/NADH-dependent reduction of INT to formazan. About 15 mg frozen quadriceps of WT and ERRα^3SA^ mice were homogenized with ice-cold 1X sample buffer (50 μl per mg tissue) and left on ice with agitation for 5 min followed by centrifugation at 15,000 g for 5 min. Briefly, 20 μl supernatants of each sample were added in a 96-well plate in duplicate, mixed with either 50 μl control solution or 50 μl reaction solution, and incubated in a 37 °C humidified incubator (no CO_2_) for 60 min. The colorimetric reaction was read at 492 nm on the Azure Ao absorbance microplate reader.

### 2.12 Lactate dehydrogenase (Ldh) assay and Ldh isoenzyme composition determination

Total Ldh activity was measured using a Lactate Dehydrogenase (LDH) Assay Kit (cat. no E-107; Biomedical Research Service). The method is based on the reduction of the tetrazolium salt INT in a NADH-coupled enzymatic reaction to formazan. Briefly, protein lysates (20 μl at 0.03 μg/μl) from skeletal muscle (gastrocnemius and soleus) and quadriceps of WT and ERRα^3SA^ mice prepared as described for immunoblotting were added in a 96-well plate, mixed with 50 μl LDH Assay buffer, and incubated in a 37 °C non-humidified incubator (no CO_2_) for 30 min. The colorimetric reaction was read at 492 nm on the Azure Ao absorbance microplate reader.

Specific Ldh isoenzyme composition and activity was determined using a Lactate Dehydrogenase (LDH) Staining Kit (cat. no E-106; Biomedical Research Service). Ldh is composed of four subunits, consisting of Ldha (type M, muscle) and/or Ldhb (type H, heart), giving rise to five potential isoenzymes. The method is based on the separation of the Ldh isoenzymes by non-denaturing agarose gel electrophoresis followed by enzymatic reduction of a tetrazolium salt to a formazan product exhibiting a dark blue color whereby its intensity is proportional to Ldh isoenzyme activity. Briefly, 12 μl protein lysates at 3 μg/μl and 2 μg/μl for skeletal muscle (gastrocnemius and soleus) and quadriceps, respectively, of WT and ERRα^3SA^ mice prepared as described for immunoblotting were mixed with 3 μl Loading Solution and loaded on a mini agarose gel apparatus. Following Ldh isoenzyme separation, mini agarose gels placed in casting trays were covered in 6 ml LDH Staining Solution, wrapped with aluminum foil to prevent evaporation, and incubated in a 37 °C humidified incubator (no CO_2_) at which reaction times were initiated. Dark blue Ldh bands were detected in skeletal muscle and quadricep lysates following a reaction time of 30 min and 1 h, respectively, and images were acquired using a ChemiDoc MP imaging System (Bio-Rad). Densitometric analyses of Ldh stained bands were performed using the open-source image analysis software Fiji (version 2.9.0/1.53t) [35].

### 2.13 LTT

For the lactate tolerance test, mice were transferred from cages with corn chip to wood chip bedding from 9:30 am to 3:30 pm. Mice were intraperitoneally injected with 2 mg/g body weight of freshly made L-lactic (cat. no. 71718; Sigma) after 6 h food deprivation. Blood was collected from the tail vein at 0, 15, 30, 60, 90 and 120 min for determination of glucose or lactate levels using the OneTouch Ultra®2 glucose meter (LifeScan) and the Lactate Scout meter (Lactate.com), respectively. Tissues were collected 4 h post lactate injection.

### 2.14 Histology

For immunohistochemistry (IHC) staining of succinate dehydrogenase (SDH), fresh muscles were snap frozen in Tissue-Tek O.C.T Compound (cat. no. 1437365; Thermo Fisher Scientific) followed by frozen sectioning at the Histology Core Facility of the Goodman Cancer Institute (GCI). Cryo-sections were thawed at room temperature (RT) and incubated for 90 min in incubation medium (100 ml 0.2 M phosphate buffer, 2.7 g sodium succinate, 100 mg Nitro blue tetrazolium chloride) placed in a glass coplin slide staining jar and then rinsed in PBS. Stained sections were then fixed in 10 % formalin-PBS solution for 5 min at RT followed by washing in 15 % alcohol for 5 min. Slides were mounted with Shandon™ Immu-Mount™ (cat. no. FS9990402; Thermo Fisher Scientific) and sealed. Slides were scanned using Aperio ScanScope XT and viewed by Aperio ImageScope (version 12.4.3.5008; Leica Biosystems).

### 2.15 Immunofluorescence

Cryo-sections were thawed at RT and fixed in 3 % paraformaldehyde in PBS for 20 min, followed by washing with three changes of PBS for 5 min each. Specimens were blocked for 1 h at room temperature with UltraCruz® Blocking Reagent (cat. no. sc-516214; Santa Cruz Biotechnology) then incubated at 4 °C overnight with primary antibodies: Laminin (1:200; cat. no. L9393; Sigma), or CD31 (1:50; cat. no. 550274; BD Pharmingen). Sections were washed with three changes of PBS for 5 min each and then incubated at RT for 1 h in a dark chamber with secondary antibodies: Donkey anti-Rabbit IgG (H + L) conjugated with Alexa Fluor 488 (1:1000; cat. no. A-21206; Invitrogen), Goat anti-Rat IgG (H + L) conjugated with Alexa Fluor 555 (1:1000; cat. no. A-21434; Invitrogen). Sections were washed with three changes of PBS for 5 min each and immediately mounted with ProLong Glass Antifade Mountant with NucBlue Stain (cat. no. P36981; Invitrogen). Images were collected using LSM 800 Confocal Microscope at Advanced BioImaging Facility (ABIF) of McGill university and processed using Zeiss Zen software (version 3.1).

For immunofluorescence (IF) staining of myosin heavy chain (MyHC) isoforms, muscles were rapidly snap-frozen with OCT embedding media in 2-methylbutane for 50 s. Then, IF was performed as previously described [36] with some modifications. Briefly, fresh (no fixation) cryo-sections (10 μm) of mouse quadriceps were blocked with 4 drops of M.O.M. IgG blocking solution (cat. no. MKB-2213-NB, Novus) in 2 ml of PBS 1X for 1 h at room temperature. Then, muscle sections were briefly washed twice with 150 μL of PBS 1X for 2 min. A solution with all the primary antibodies in PBS containing 0.5 % of bovine serum albumin (BSA, cat. no. 200-095-CG, Multicell) was then prepared (cat. no. SC-71 (IgG1, supernatant, 1:100 dilution, Developmental Studies Hybridoma Bank) specific for MyHC-2A; cat. no. BF-F3 (IgM, supernatant, 1:100 dilution, Developmental Studies Hybridoma Bank) specific for MyHC-2B; cat. no. L9393 (IgR 3.5 μg/ml dilution, Sigma) specific for laminin to visualize sarcolemma). Sections were incubated for 1 h at 37 °C in humid chambers with primary antibodies. After, sections were washed three times (5 min each) with PBS 1X. Then, sections were incubated for 1 h at 37 °C in humid chambers in the dark with a solution with the three different secondary antibodies, diluted in PBS containing 0.5 % BSA and 5 % goat serum (cat. no. G9023-10 ML, Sigma): goat anti-mouse IgG1, conjugated with DyLight488 fluorophore (cat. no. 115-547-185; Jackson Immunoresearch; to bind to SC-71) at dilution 1:100; goat anti-mouse IgM, conjugated with DyLight594 fluorophore (cat. no. 115-587-020; Jackson Immunoresearch to bind to BF-F3) at dilution 1:100; goat anti-rabbit IgG (H + L) Highly Cross-Adsorbed Secondary Antibody, Alexa Fluor™ 647 (cat. no. A-21245, Thermo Fisher to bind to laminin) at dilution 2 μg/ml. Next, sections were then washed three times (5 min each) with PBS 1X. Sections were mounted with Immu-Mount™ (cat. no. 9990402, Thermo scientific). Pictures were collected with an epifluorescence Axiovert 1 Zeiss microscope. Single-color images were merged to obtain a whole muscle reconstruction with Zen Blue software using Stitching method and a reference image for the background of each muscle. Then, specific muscle fibers were counted manually.

### 2.16 Mitochondrial DNA to nuclear DNA (mtDNA/nDNA) ratio

The relative number of mitochondria in mouse quadriceps muscle was estimated by measuring the ratio of mtDNA:nDNA. Genomic DNA was extracted using the DNeasy blood and tissue kit (cat. no. 69506; QIAGEN) with RNase A being used to degrade cellular RNA. DNA was quantified and used for qPCR amplification. The relative amounts of mtDNA and nDNA were quantified using primers specific for mitochondrial gene 16 S ribosomal RNA (16 S rRNA; forward, 5′-CCGCAAGGGAAAGATGAAAGAC-3′; and reverse, 5′-TCGTTTGGTTTCGGGGTTTC-3′) and the nuclear gene Hexokinase 2 (HK2; forward, 5′-GCCAGCCTCTCCTGATTTTAGTGT-3′; and reverse, 5′-GGGAACACAAAAGACCTCTTCTGG-3′).

### 2.17 Ion pairing LC-MS/MS metabolite profiling

Skeletal muscle was collected 3.5 h post endurance running and snap-frozen in liquid nitrogen followed by pulverization in liquid nitrogen. 10 ± 1 mg tissue were weighed per sample and subjected to bead beating (Eppendorf Tissue-lyser) together with 1,140 μl of 50 % methanol (MeOH) and 660 μl of acetonitrile (ACN) for 2 min at 30 Hz. Lipids were partitioned through the addition of 1,800 μl of cold dichloromethane and 900 μl of cold H2O. Samples were kept as cold as possible during extraction. The upper aqueous layer was then removed and dried by vacuum centrifugation (LabConco) with sample temperature maintained at −4 °C. Each sample was individually resuspended in 50 μl of cold water and immediately subjected to LC-MS analysis. Metabolite separation was achieved by using a 1290 UPLC equipped with a Zorbax Extend C18 column 1.8 μm, 2.1 × 150 mm^2^ with guard column 1.8 μm, 2.1 × 5 mm^2^ (Agilent Technologies). The chromatographic gradient started at 100 % mobile phase A (97 % water, 3 % methanol, 10 mM tributylamine, 15 mM acetic acid, 5 μM medronic acid) for 2.5 min, followed with a 5-min gradient to 20 % mobile phase C (methanol, 10 mM tributylamine, 15 mM acetic acid, 5 μM medronic acid), a 5.5-min gradient to 45 % C and a 7-min gradient to 99 % C at a flow rate of 0.25 ml min^−1^. This was followed by a 4-min hold time at 100 % mobile phase C. The column was restored by back-washing with 99 % mobile phase D (90 % ACN) for 3 min at 0.25 ml min^−1^, followed by increase of the flow rate to 0.8 ml min^−1^ over 0.5 min and a 3.85-min hold, after which the flow rate was decreased to 0.6 ml min^−1^ over 0.15 min. The column was then re-equilibrated at 100 % A over 0.75 min, during which the flow rate was decreased to 0.4 ml min^−1^, and held for 7.65 min. One minute before the next injection, the flow was brought back to forward flow at 0.25 ml min^−1^. The column temperature was maintained at 35 °C. Eluants were detected by a triple quadrupole mass spectrometer (6470 QQQ Agilent) Dynamic multiple reaction monitoring (dMRM) was used for the detection of 269 metabolites (207 in range curves, 34 validated (not in calibration curves), 28 compounds from the Agilent Ion pairing library, not fully validated by the facility. Retention time and MRM transitions were optimized using authentic standards for all validated compounds. Metabolite saturation levels were determined from external curves for the most commonly detected metabolites of central carbon metabolism. Area under the curve for each sample and metabolite analyzed and ensured to be below the saturation limit for those metabolites where range curves were available. No corrections or allowances were made for ion suppression effects. Relative abundance of metabolites was normalized to tissue weight.

### 2.18 Statistics and reproducibility

GraphPad Prism 9 were used to generate graphs and for statistical analyses. The specific statistical tests and definition of error bars are denoted in the corresponding figure legend. For all data, differences were considered significant when p < 0.05. “n” values represent individual mice or biological replicates. Sample size was not pre-specified statistically. Panels shown without biological replicates are representative of independent experiments.

### 2.19 Data availability

ERRα liver and skeletal muscle ChIP-sequencing data performed on ERRα^3SA^ and WT mouse littermates have been deposited in NCBI's Gene Expression Omnibus (GEO) under the accession number GEO: GSE219046. Muscle RNA-sequencing data of WT and ERRα^3SA^ mice are under the accession number GEO: GSE182000. RNA-sequencing data of exercise-induced muscle genes was from the original study for GSE97718.

## 3 Results

### 3.1 Exercise stimulates the expression of ERRα protein

**Figure 1:**
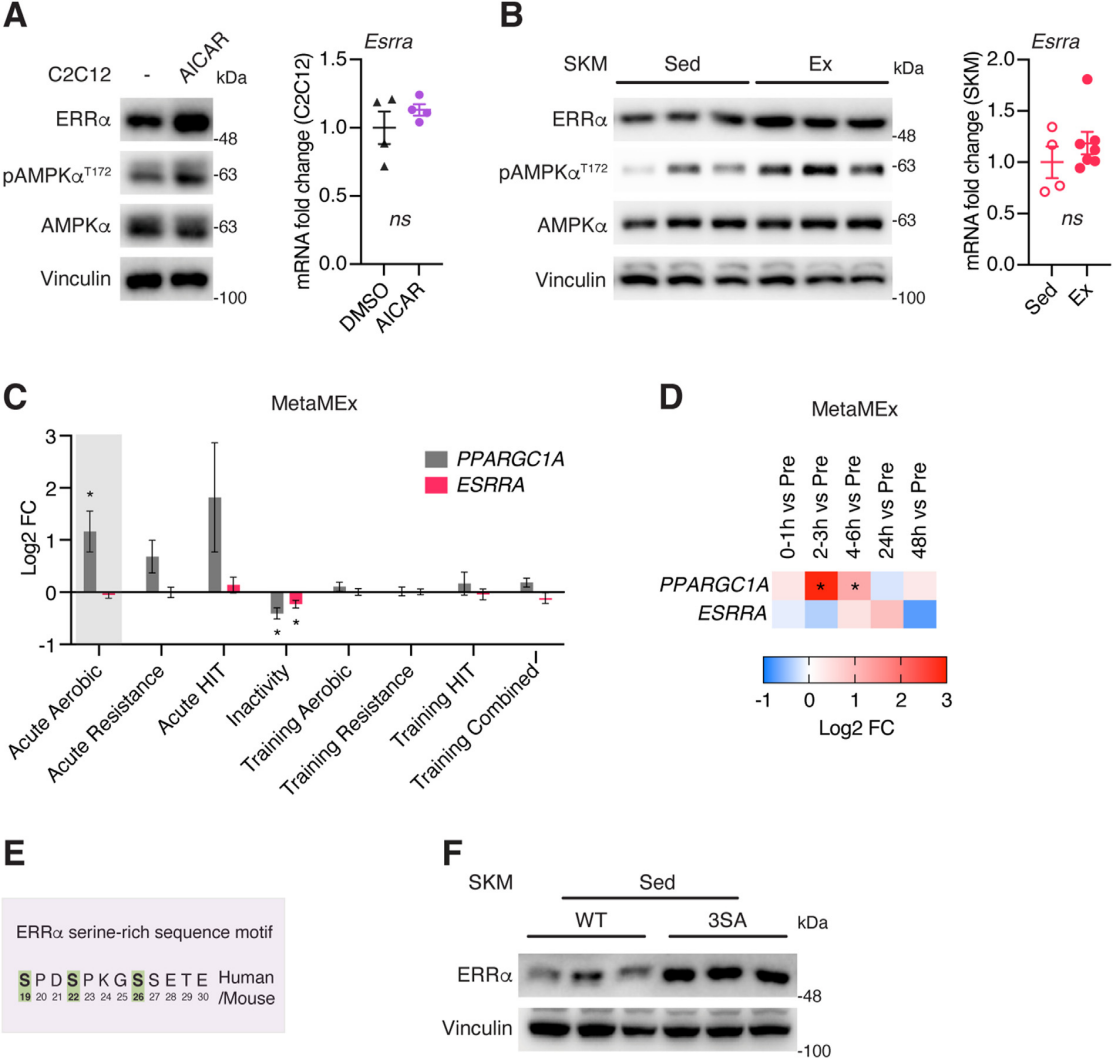
**Exercise induces ERRα protein expression.** (A) Immunoblots (left) and RT-qPCR (right, n = 4) examination of C2C12 muscle cells treated with 1 mM AICAR for 6 h. (B) Immunoblots (left, each lane represents one mouse, n = 3) and RT-qPCR (right, n = 4–7) examination of skeletal muscle (SKM) from sedentary (Sed) or exercised (Ex) mice. (C) Transcript profiles of *PPARGC1A* and *ESRRA* across 66 human skeletal muscle transcriptomic studies from the online tool MetaMEx. (D) *PPARGC1A* and *ESRRA* mRNA expression changes among the acute aerobic exercise datasets in (B) subdivided into different post recovery times relative to unexercised (Pre) control muscle. (E) Schematic of ERRα serine-rich sequence motif. (F) Immunoblot of ERRα protein in skeletal muscles of WT and ERRα^3SA^ mice. Each lane represents one mouse, n = 3. Data (A,B) are presented as means ± SEM, unpaired two-tailed Student's t test. ns: not significant. Data (C,D) are presented as means ± variance (95 % confidence intervals) or log2 fold-changes, ∗p < 0.05, Benjamini-Hochberg adjusted p-values.

We first aimed to explore how exercise enhances ERRα functional activity. Muscle contraction usually occurs with ATP turnover, AMPK activation, calcium flux, and oxygen pressure [22]. AMPK agonists by conferring multiple exercise-like changes in signalling, transcription and metabolism are considered as “exercise-mimetics” [7,37]. We observed accumulated ERRα protein in C2C12 muscle cells treated with AMPK activator AICAR, as well as in skeletal muscle of exercised mice following a 3.5 h recovery period (Figure 1A,B). An independent experiment confirmed that complete food restriction during the entire course of the exercise regimen did not prevent exercise-induced ERRα protein accumulation (Suppl. Fig. 1A), thus excluding possible post-feeding effects on ERRα induction. Notably, the augmented ERRα protein levels in response to AICAR and exercise were independent of transcriptional changes (Figure 1A,B). While transcription of the ERRα-encoded gene *Esrra* has been shown in a limited number of reports to be upregulated by exercise in both mice and humans [[21], [22], [23]], interrogation of a recent human transcriptome meta-analysis integrating 66 public exercise response datasets accessible through the web tool MetaMEx (www.metamex.eu; [38]) established that *ESRRA* is generally not transcriptionally activated in skeletal muscle by acute aerobic exercises (Figure 1C,D). In contrast, levels of *PPARGC1A* encoding PGC-1α are strongly elevated following a 2–3 h recovery period and to a lesser extent at 4–6 h post an acute aerobic exercise (Figure 1C,D). PGC-1α activity is well-known to be enhanced by endurance exercise and serves as an essential ERRα co-activator [[39], [40], [41], [42]]. Clearly, mechanisms other than PGC-1α coactivation of ERRα including ERRα protein stabilization underly the stimulatory actions of exercise on ERRα activity. We have previously identified three serine residues in ERRα central to a highly-conserved, strictly-regulated sequence motif, S(19)PDS(22)PKGS(26)SETE (Figure 1 E), that dictates ERRα cellular localization, protein stability and biological activity [28]. ERRα^3SA^ mice harboring mutations at these three serine residues result in a phospho-deficient genetic model exhibiting robust ERRα protein stabilization, as observed in skeletal muscle (Figure 1 F), essentially mimicking the inducible effects of exercise on ERRα protein level in this tissue. It is worth noting that phospho-ERRα S19 levels were not found responsive to AICAR or exercise (Suppl. Figs. 1B and C), and due to a lack of antibodies targeting the other two serines (S22 and S26), it is not clear whether AICAR- or exercise-induced increases in ERRα protein levels involve these specific residues.

### 3.2 ERRα genetic activation enhances exercise capacity

**Figure 2:**
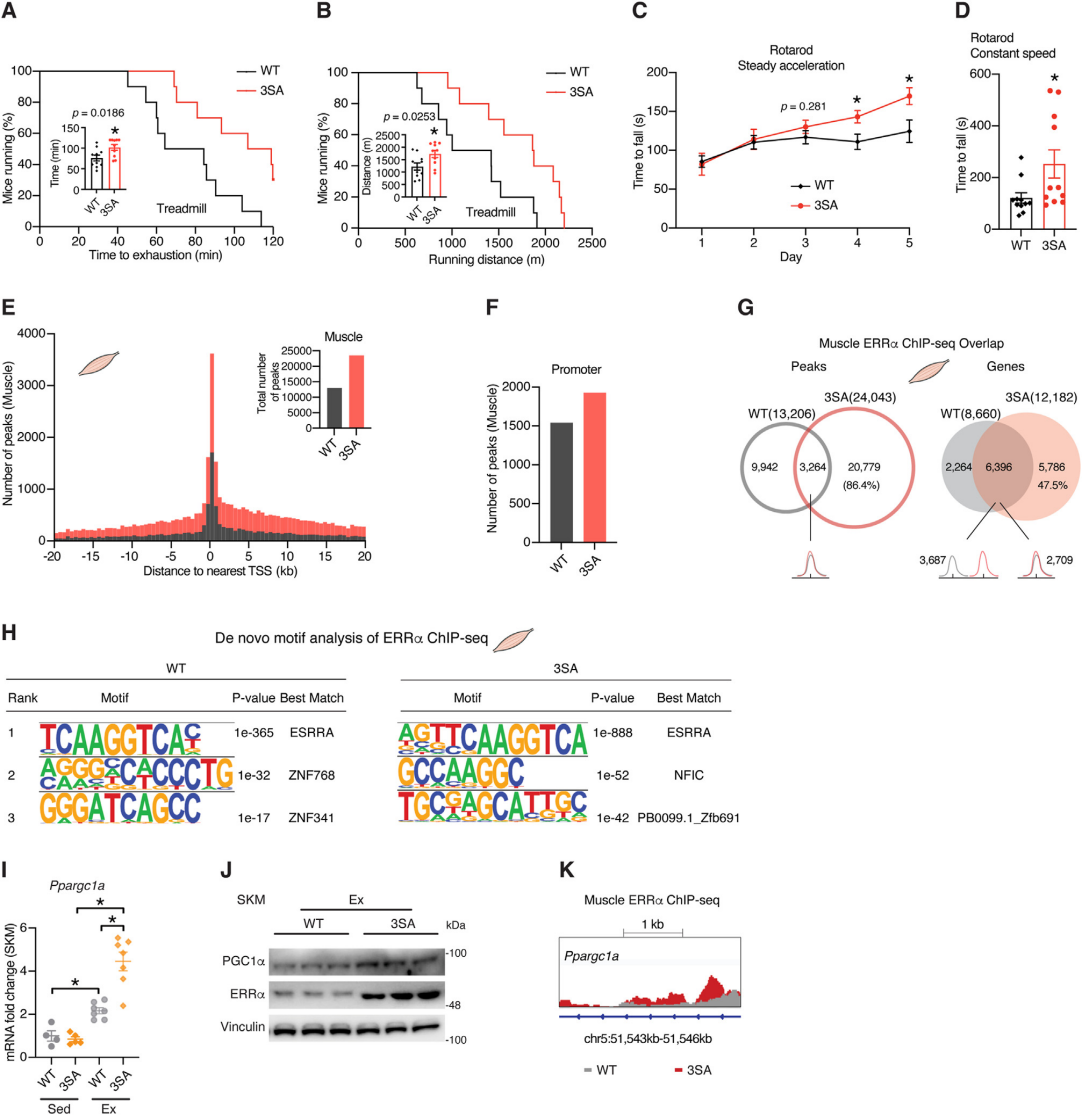
**ERRα genetic activation enhances exercise capacity.** (A) Treadmill exhaustion test performed in WT and ERRα^3SA^ mice (n = 10). Percentage of mice running at the indicated time. Inner histogram shows the mean duration of the run. (B) Treadmill exhaustion test performed in WT and ERRα^3SA^ mice (n = 10). Percentage of mice running at indicated distances. Inner histogram shows the mean running distance. (C, D) Motor coordination ability was evaluated in WT and ERRα^3SA^ mice (n = 11) as the time to fall from a rotarod either under (C) steady acceleration (4–40 rpm for 300 s) for five consecutive days or at (D) constant speed (20 rpm for 600s). (E) Distribution of skeletal muscle ERRα ChIP-seq peaks ± 20 kb relative to the transcription start site (TSS) of the nearest gene identified in ERRα^3SA^ and WT littermates. Inner histogram shows the total number of peaks bound by ERRα within ±20 kb of TSS. (F) Number of promoter-annotated muscle ERRα ChIP-seq peaks. (G) Overlap of ERRα ChIP-seq binding peaks (left) and target genes (right) within ±20 kb of TSS identified in the skeletal muscle of ERRα^3SA^ and WT littermates. (H) De novo motif computational discovery of ERRα-bound sequences (within ±20 kb of TSS) in the skeletal muscle of ERRα^3SA^ and WT littermates. (I) *Ppargc1a* mRNA levels in skeletal muscle of WT and ERRα^3SA^ mice in the sedentary state or post the treadmill exhaustion test, n = 4–7. (J) Immunoblots of skeletal muscle PGC-1α proteins in WT and ERRα^3SA^ sedentary mice and post the treadmill exhaustion test. Each lane represents one mouse, n = 3. (K) WT and ERRα^3SA^ muscle ChIP-seq tracks at *Ppargc1a*. Data are presented as means ± SEM, ∗p < 0.05, unpaired two-tailed Student's t test (A-D,I).

To test potential consequences of the loss of amino terminal domain phosphorylation on ERRα functional activity in muscle, we first subjected the mice to a treadmill exhaustion test and found that ERRα^3SA^ mice significantly outperformed their WT littermates, reflected by increased running time and distance, as well as the total work performed (Figure 2 A,B; Suppl. Fig. 2 A). By using both steady-accelerating and constant-speed rotarod we further confirmed enhanced motor coordination ability of ERRα^3SA^ mice (Figure 2C,D), which are independent of alterations in muscle strength (Suppl. Fig. 2 B).

Given that dephosphorylation retains ERRα in the nucleus [28], we next sought to investigate whether it impacts the genome-wide interaction of ERRα with chromatin. To this end, we interrogated skeletal muscle and liver of ERRα^3SA^ and WT mice. ERRα ChIP followed by high-throughput sequencing (ChIP-seq) revealed an over 1.8-fold increase in the number of ERRα-bound peaks by 3SA mutation within ±20 kb of gene transcription start sites (TSSs), highlighting increased ERRα recruitment to gene promoter regions in skeletal muscle (Figure 2 E,F; Suppl. Table 1). A similar although less pronounced pattern was also observed in the liver (Suppl. Fig. 2C,D; Suppl. Table 2). Notably, the 3SA mutation greatly remodeled the ERRα cistrome, especially in the muscle, with over 85 % peaks and around 50 % target genes identified in ERRα^3SA^ muscle being unique (Figure 2 G; Suppl. Fig. 2 E; Suppl. Tables 1 and 2). De novo motif analyses identified the consensus ERR response element (ERRE) as the most enriched motif in ERRα-bound segments within ±20 kb of TSSs in all conditions, with the enrichment becoming much more significant upon 3SA mutation (Figure 2H; Suppl. Fig. 2 F), highlighting dephosphorylation as a key trigger to unmask previously unknown ERRα regulated targets.

Interestingly, improved muscle performance of ERRα^3SA^ mice was found accompanied by a greater induction of PGC-1α (Figure 2I,J; Suppl. Fig. 2 G,H). ERRα chromatin occupancy at *Ppargc1a* was simultaneously increased in ERRα^3SA^ muscle (Figure 2 K), indicating a direct-regulatory and mutual-activating partnership between ERRα and PGC-1α. By contrast, *Ppargc1b* expression was downregulated by exercise and remained unaltered between genotypes (Suppl. Fig. 2 I). Moreover, ERRα^3SA^ mice displayed elevated muscle activation of AMPK when subjected to exercise (Suppl. Fig. 2 J), suggesting a feedforward loop.

### 3.3 Improved lactate homeostasis during exercise by ERRα

**Figure 3:**
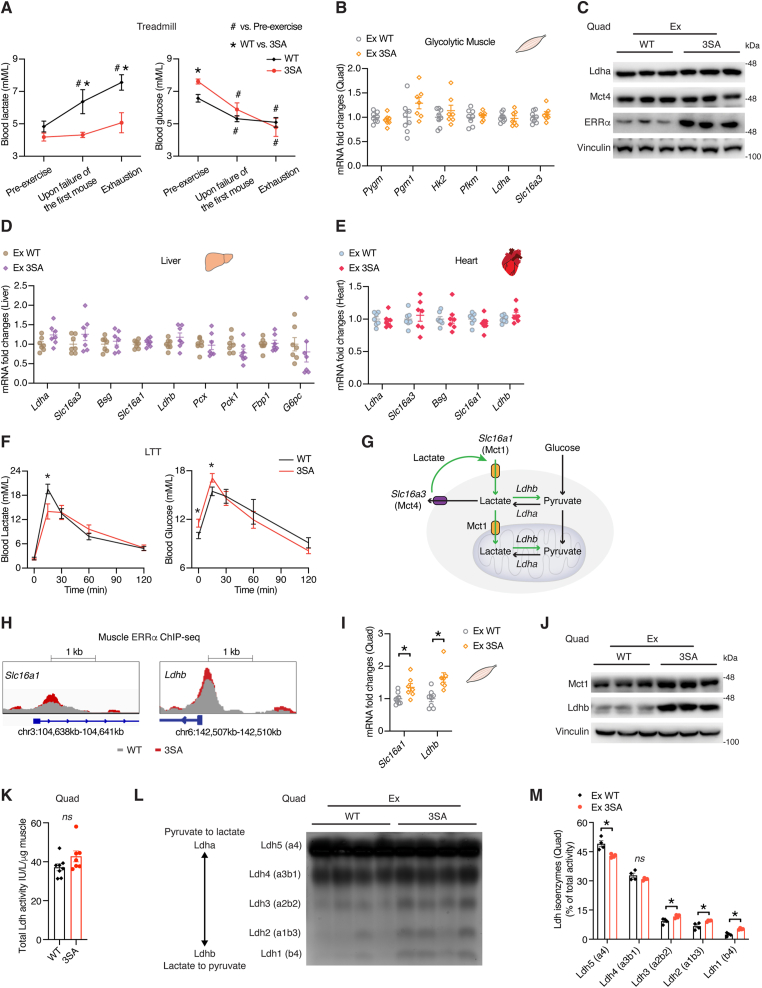
**ERRα promotes lactate homeostasis during exercise.** (A) Blood lactate (left) and glucose (right) concentrations during the treadmill exhaustion test, n = 7–12. (B) mRNA levels of the indicated genes in glycolytic quadriceps (Quad) of WT and ERRα^3SA^ mice post the treadmill exhaustion test, n = 7–8. (C) Immunoblots of quadriceps of WT and ERRα^3SA^ mice post the treadmill exhaustion test. Each lane represents one mouse, n = 3. (D,E) mRNA levels of the indicated genes in liver (D) and heart (E) of WT and ERRα^3SA^ mice post the treadmill exhaustion test, n = 7. (F) Blood lactate (left) and glucose (right) concentrations during the lactate tolerance test (LTT), n = 6. (G) Illustration of lactate metabolism in muscle. (H) WT and ERRα^3SA^ muscle ChIP-seq tracks at *Slc16a1* and *Ldhb* gene loci. (I,J) mRNA (I, n = 7–8) and protein (J, each lane represents one mouse, n = 3) levels of Mct1 and Ldhb in quadriceps (Quad) of WT and ERRα^3SA^ mice post the treadmill exhaustion test. (K) Total LDH activity in quadriceps (Quad) of WT and ERRα^3SA^ mice post the treadmill exhaustion test (n = 7–8). (L) Non-denaturing agarose gel electrophoresis determination of Ldh isoenzyme composition in quadriceps (Quad) of WT and ERRα^3SA^ mice post the treadmill exhaustion test (each lane represents one mouse, n = 4). (M) Quantification of individual Ldh isoenzymes identified in (L) expressed as percent of total Ldh activity. Data are presented as means ± SEM, ∗, #p < 0.05, unpaired two-tailed Student's t test (A,B,D-F,I,K,M). ns: not significant.

Another striking finding from the treadmill test is that ERRα^3SA^ mice maintained steady blood lactate levels throughout the running, while WT mice exhibited a continuous rise in circulating lactate levels generating a 33 % reduction in lactate levels in ERRα^3SA^ mice at exhaustion (Figure 3A). Lactate production from muscle fibers and its consequent secretion into blood reflects muscle glycolytic rate, a strong indicator of exercise performance. Despite similar inverse correlation rates between blood lactate levels at exhaustion and exercise tolerance parameters between WT and ERRα^3SA^ mice, the lower slopes observed for ERRα^3SA^ mice signify that their increased fitness is directly related to their ability to maintain lower blood lactate levels (Suppl. Fig. 3 A,B). ERRα^3SA^ mice presented significantly higher resting but comparable post-exercise blood glucose levels (Figure 3 A), implying increased glucose uptake. Key genes implicated in glycogenolysis, glycolysis, lactate generation and export showed similar levels in glycolytic quadriceps (Quad) of WT and ERRα^3SA^ mice, suggesting that diminished lactate production is not an underlying factor (Figure 3B,C). Liver and heart play important roles in buffering blood lactate, either using it for hepatic gluconeogenesis or directly burning it as a cardiac fuel [43]. Neither hepatic or cardiac genes involved in lactate utilization showed any differences (Figure 3D,E), indicating that these tissues are not primarily responsible for the improved whole-body lactate homeostasis of ERRα^3SA^ mice. A lactate tolerance test (LTT), revealing 28 % lower lactate levels in ERRα^3SA^ mice post 15 min of its administration, further confirmed an enhanced ability of ERRα^3SA^ mice to regulate blood lactate levels and its independence of hepatic glucose production from lactate (Figure 3 F). During exercise, lactate serves as the link between glycolysis and oxidative phosphorylation (OxPhos), lactate produced through glycolysis by fast-twitch muscle fibers could also be used in the mitochondria of oxidative fibers as energy fuel (Figure 3 G). 3SA mutation increased ERRα recruitment to muscle *Slc16a1* (*Mct1*, encoding monocarboxylate transporter 1) and *Ldhb* (encoding lactate dehydrogenase B) (Figure 3H), which together mediate lactate import and conversion into pyruvate to refuel the mitochondria. Indeed, oxidative skeletal muscles of ERRα^3SA^ mice displayed increased Mct1 and Ldhb levels both post exercise and when stimulated with exogenous lactate (Suppl. Fig. 3C–E), facilitating lactate reutilization. Remarkably, increases in *Mct1* and *Ldhb* mRNAs and corresponding proteins were also observed in glycolytic quadriceps of ERRα^3SA^ mice (Figure 3I,J), suggesting a potential switch to oxidative myofiber. Consistently, while no change in total lactate dehydrogenase (Ldh) activity was found, both glycolytic and oxidative muscles of ERRα^3SA^ mice displayed altered Ldh isoenzyme composition marked by increased Ldh1 (Ldhb) and decreased Ldh5 (Ldha) activities, underscoring their enhanced capacity to lower lactate levels via its oxidation to pyruvate (Figure 3 K-L; Suppl. Fig. 3F–H). Interestingly, transgenic mice overexpressing Ldhb in muscle displayed increased exercise performance [44].

### 3.4 ERRα promotes oxidative muscle fiber type and angiogenesis

**Figure 4:**
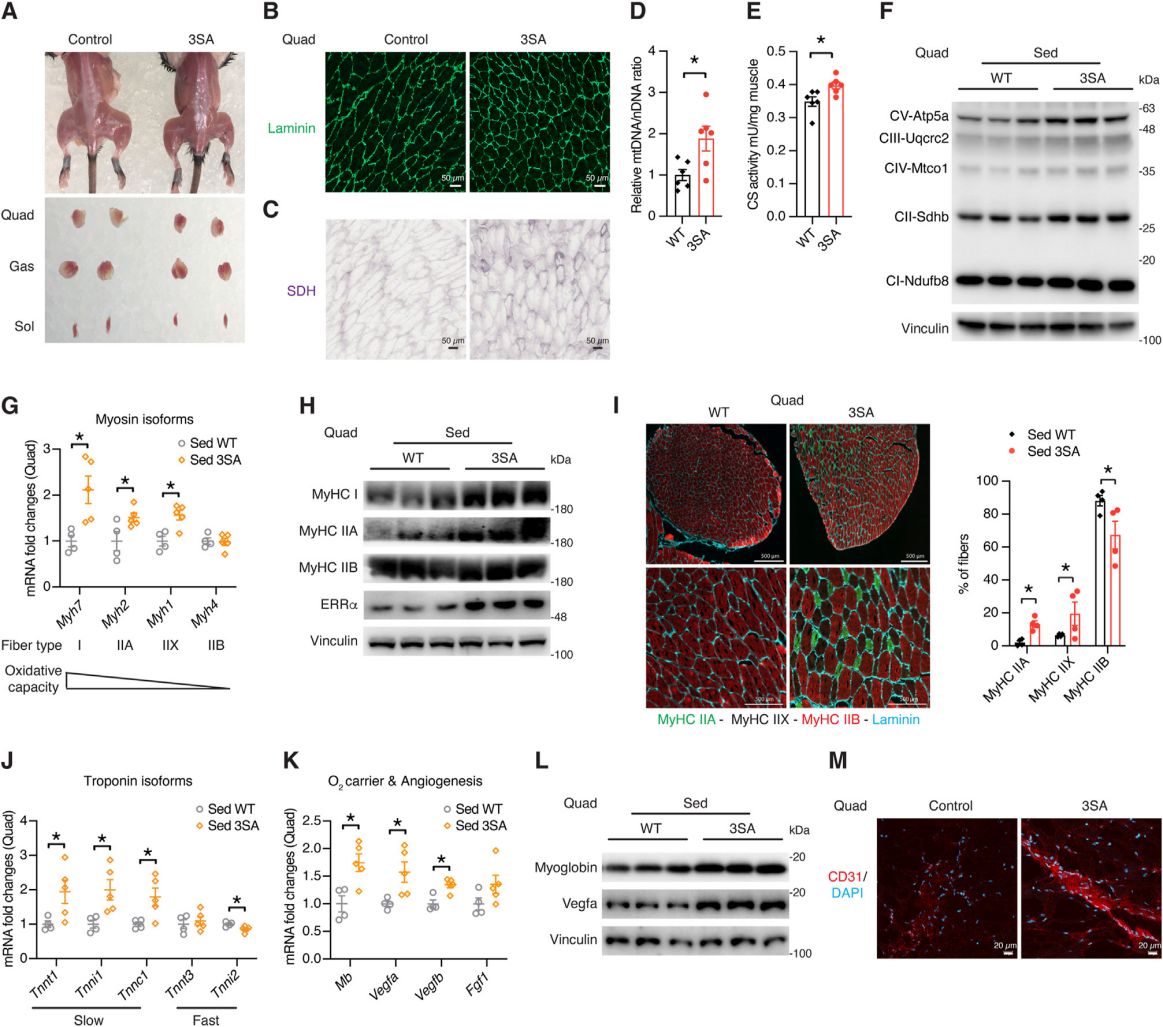
**ERRα promotes oxidative muscle fibers and angiogenesis.** (A) Representative photographs showing muscle appearances of ERRα^3SA^ mice and their littermate controls. Quad: quadriceps; Gas: gastrocnemius; Sol: soleus. (B,C) Representative Laminin stained (B) and succinate dehydrogenase (SDH) stained (C) cross-sections of quadriceps from ERRα^3SA^ mice and their littermate controls. Scale bar represents 50 μm. (D) Relative levels of mitochondrial DNA (mtDNA) to nuclear DNA (nDNA) of quadriceps from ERRα^3SA^ and WT littermates (n = 7–8). (E) Citrate synthase (CS) activity in quadriceps from ERRα^3SA^ and WT littermates (n = 6). (F) Immunoblots of mitochondrial Oxphos proteins in quadriceps from WT and ERRα^3SA^ mice. Each lane represents one mouse, n = 3. (G,H) mRNA (G, n = 4–5) and protein (H, each lane represents one mouse, n = 3) levels of genes encoding distinct myosin isoforms in quadriceps of ERRα^3SA^ and WT littermates. (I) Representative MyHC IIA and IIB immunofluorescence staining of cross-sections of quadriceps from ERRα^3SA^ mice and their littermate controls. MyHC IIX appears as unstained. Below, enlarged magnification for each muscle. Scale bar represents 500 μm. Quantification of fibers expressed as percentage of fibers is indicated for each MyHC. (J) mRNA levels of genes encoding distinct troponin isoforms in quadriceps of ERRα^3SA^ and WT littermates, n = 4–5. (K,L) mRNA (K, n = 4–5) and protein (L, each lane represents one mouse, n = 3) levels of myoglobin and angiogenesis genes in quadriceps of ERRα^3SA^ and WT littermates. (M) Quadriceps from ERRα^3SA^ and control mice were fluorescently stained for CD31. Nuclei were counterstained with DAPI. Scale bar represents 20 μm. Data are presented as means ± SEM, ∗p < 0.05, unpaired two-tailed Student's t test (D,E,G,I-K).

Despite unchanged muscle mass (Suppl. Fig. 4A), ERRα^3SA^ muscles displayed a redder appearance, particularly evident in quadriceps (Figure 4 A). Morphometric analyses with Laminin staining and histochemical analysis for succinate dehydrogenase (SDH) activity demonstrated that ERRα^3SA^ quadriceps have smaller myofibers and increased muscle respiratory capacity (Figure 4B,C), which are all consistent with a more oxidative muscle phenotype. Indeed, quadriceps of ERRα^3SA^ mice were found to possess about two-fold more mitochondrial DNA (mtDNA) contents in comparison with their WT littermates (Figure 4D), likely attributed in part by increased *Cmpk2* levels (Suppl. Fig. 4B), which encodes a rate-limiting enzyme supplying deoxyribonucleotides for mtDNA synthesis. Consistently, higher citrate synthase (CS) activity was observed in ERRα^3SA^ quadriceps (Figure 4E), signifying their increased mitochondrial biogenesis, in parallel with elevated detection of mitochondrial OxPhos proteins (Figure 4F). Further examination of a spectrum of myofiber markers revealed upregulated expression of genes characterizing oxidative and slow-twitch contractile apparatus together with increased oxidative fiber type specificity in quadriceps of ERRα^3SA^ mice (Figure 4G–J), in line with enhanced occupancy of ERRα^3SA^ protein at these genes (Suppl. Fig. 4C). The oxygen carrier myoglobin and angiogenesis genes that are crucial for muscle adaptations to elevated oxidative demand were simultaneously upregulated in quadriceps of ERRα^3SA^ mice (Figure 4K,L). Further morphometric analyses with CD31, a marker routinely used to detect angiogenesis and capillaries, confirmed increased vasculature density of ERRα^3SA^ muscle that would facilitate their oxygen replenishment (Figure 4M). These results together uncovered ERRα as a crucial regulator triggering myofiber aerobic transformation without exercise training.

### 3.5 Skeletal muscle ERRα augments mitochondrial fat oxidation

**Figure 5:**
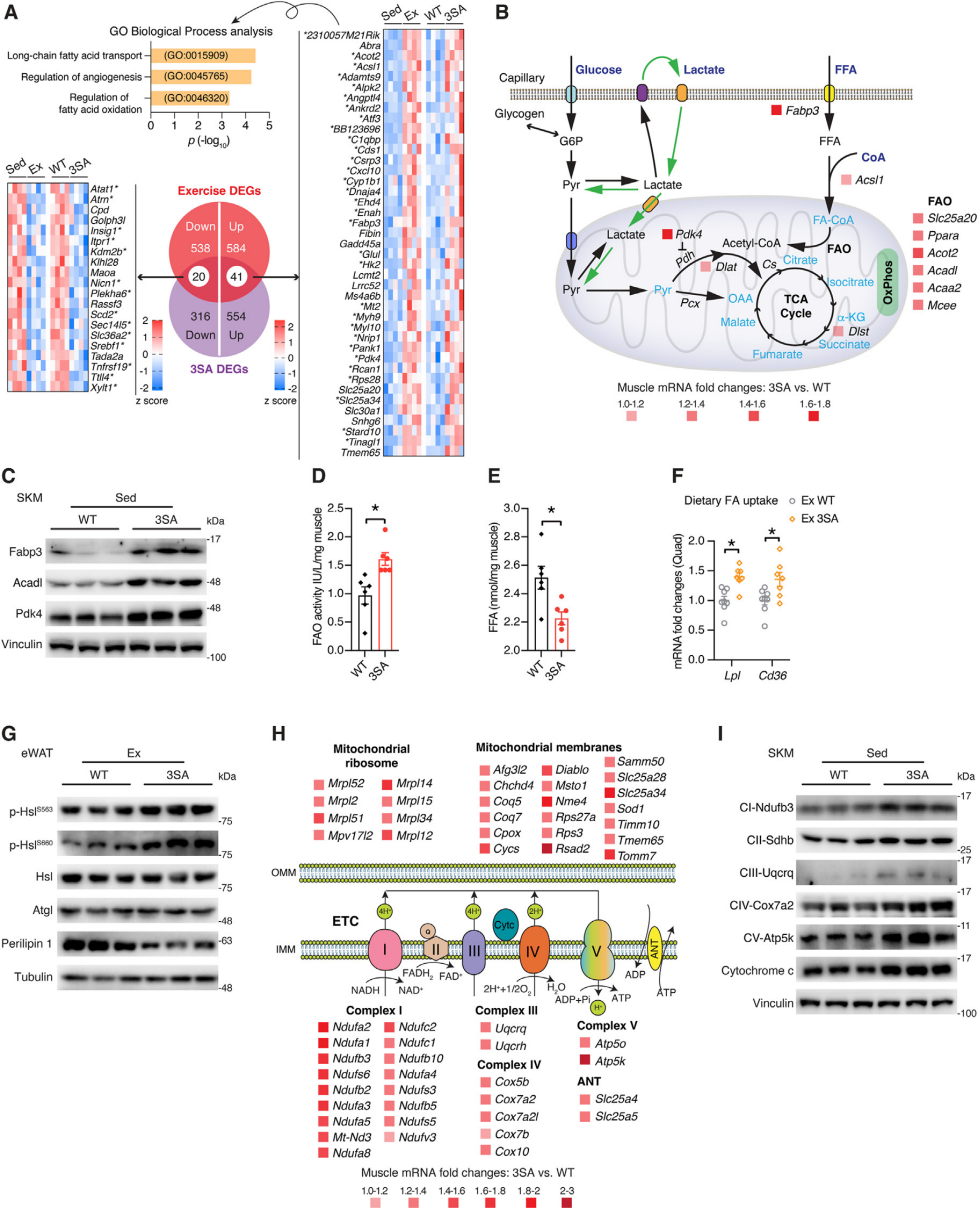
**Skeletal muscle ERRα enhances mitochondrial fat oxidation.** (A) Venn diagram, heatmap representation, and pathway enrichment analysis of skeletal muscle ERRα^3SA^ DEGs consistently regulated by exercise (p < 0.05). Genes with an asterix denote the presence of an ERRα binding event (±20 kb TSS) identified by ChIP-seq. (B) Schematic illustration of distinct fuel metabolism that converges in mitochondria. Significantly altered genes were denoted according to muscle RNA-seq analysis ERRα^3SA^ versus WT mice (p < 0.05). Altered *Acadl* levels (p = 0.54) were confirmed by RT-qPCR validation. G6P: Glucose-6-phosphate; Pyr: Pyruvate; Pdh: Pyruvate dehydrogenase; FFA: Free fatty acid; FAO: Fatty acid oxidation; CoA: Coenzyme A; OAA: Oxaloacetate; α-KG: α-ketoglutarate; OxPhos: oxidative phosphorylation; TCA cycle: Tricarboxylic Acid cycle. (C) Immunoblots of fat oxidation proteins in skeletal muscles of WT and ERRα^3SA^ mice. Each lane represents one mouse, n = 3. (D) Muscle FAO activities of WT and ERRα^3SA^ mice post the treadmill exhaustion test, n = 6. (E) Muscle FFA contents of WT and ERRα^3SA^ mice post the treadmill exhaustion test, n = 6. (F) Relative mRNA levels of fatty acid (FA) uptake genes in quadriceps (Quad) from exercised ERRα^3SA^ and WT littermate controls, n = 7–8. (G) Immunoblots of proteins related to epididymal white adipose tissue (eWAT) lipolysis. Each lane represents one mouse, n = 3. (H) Schematic diagram of significantly upregulated genes encoding diverse mitochondrial proteins according to muscle RNA-seq analysis of ERRα^3SA^ versus WT mice (p < 0.05). (I) Immunoblots of proteins related to mitochondrial respiration in skeletal muscle from WT and ERRα^3SA^ mice. Each lane represents one mouse, n = 3. Data are presented as means ± SEM, ∗p < 0.05, unpaired two-tailed Student's t test (D–F).

ERRα 3SA phospho-mutation induced an array of 931 differentially expressed genes (DEGs, p < 0.05) in skeletal muscle [28] of which 7 % (61) consist of genes consistently modulated by exercise [45], found largely upregulated by ERRα activation and exercise, and substantially enriched in long-chain fatty acid transport, angiogenesis, and fatty acid oxidation (FAO) (Figure 5A). Among these genes, 74 % (45 of 61) were identified as direct ERRα targets (±20 kb TSS), denoted by an asterix, through cross-examination of skeletal muscle ERRα^3SA^ DEGs and ERRα ChIP-seq profiles (Figure 5A; Suppl. Fig. 5A). The major energy substrates supporting exercise are muscle glycogen, blood glucose/lactate, and fatty acid [9]. Trained muscle exhibits increased fat oxidation, which has a lower output but higher capacity than carbohydrate metabolism and is thus associated with prolonged endurance exercise [46]. Essential genes involved in fat transport and oxidation, such as *Fabp3*, *Acsl1*, *Slc25a20*, *Ppara*, *Acot2*, *Acadl*, *Acaa2*, *Pdk4*, were all elevated in ERRα^3SA^ muscle, partly contributed by increased recruitment of ERRα^3SA^ protein to these gene loci (Figure 5B,C; Suppl. Fig. 5B). In agreement, when subjected to exercise, ERRα^3SA^ mice displayed significantly higher muscle FAO enzyme activity compared with their control littermates (Figure 5D), accompanied by similar muscle glycogen levels, and significantly reduced intramuscular free fatty acid (FFA) (Figure 5E; Suppl. Fig. 5C), an indicator of active muscle. In addition, skeletal muscle can readily acquire blood FFAs, mainly derived from hydrolysis of triglycerides in chylomicrons or adipose tissue, which are essential energy sources upon sustained exercise [47]. We observed elevated muscle expression of genes encoding lipoprotein lipase (Lpl) and fatty acid transporter (FAT/Cd36) in exercised ERRα^3SA^ mice (Figure 5F). Further examination of epididymal white adipose tissue (eWAT) demonstrated accelerated lipolysis of ERRα^3SA^ mice upon exercise, reflected by increased phosphorylation of hormone-sensitive lipase (Hsl) and significantly reduced Perilipin 1 (encoded by *Plin1*), an adipose lipid droplet (LD) surface protein known to shield LDs from lipolysis (Figure 5G; Suppl. Fig. 5D). Interestingly, enhanced dietary FA uptake and adipose lipolysis in exercised ERRα^3SA^ mice occurred without alteration in circulating FFA levels (Suppl. Fig. 5E), indicating elevated muscle FFA uptake and oxidation. Acetyl-CoA generated from either glycolysis or β-oxidation converge in mitochondria to be completely oxidized through the citrate cycle (TCA cycle) and OxPhos for maximal energy generation, processes found upregulated in ERRα^3SA^ muscle by Gene Set Enrichment Analysis (GSEA, https://www.gsea-msigdb.org/gsea/index.jsp) (Suppl. Fig. 5F). Closer inspection of DEGs revealed the upregulation of a large set of genes encoding proteins involved in mitochondrial ribosome, membranes, and complex I, III, IV, V of the electron transport chain, together with adenine nucleotide translocator (ANT) in ERRα^3SA^ muscle (Figure 5H). ERRα^3SA^ proteins showed increased binding affinity at important mitochondrial genes in the muscle, suggesting direct regulation (Suppl. Fig. 5G). Immunoblotting analyses further confirmed elevated expression of OxPhos subunits and electron transporter Cytochrome c in ERRα^3SA^ muscle at the protein level (Figure 5I). Skeletal muscle mitochondrial biogenesis and turnover are highly regulated. Autophagy and mitophagy are established to play fundamental roles in maintaining mitochondrial health and organismal homeostasis during exercise [[48], [49], [50]]. In accordance with increased AMPK activation (Suppl. Fig. 2J), we observed a greater induction of autophagy/mitophagy-related genes by exercise in skeletal muscle of ERRα^3SA^ mice (Suppl. Fig. 5H). Activation of this stress response would restore exercise damage and boost endurance capacity.

### 3.6 ERRα drives alternative metabolic paths in exercising muscle

**Figure 6:**
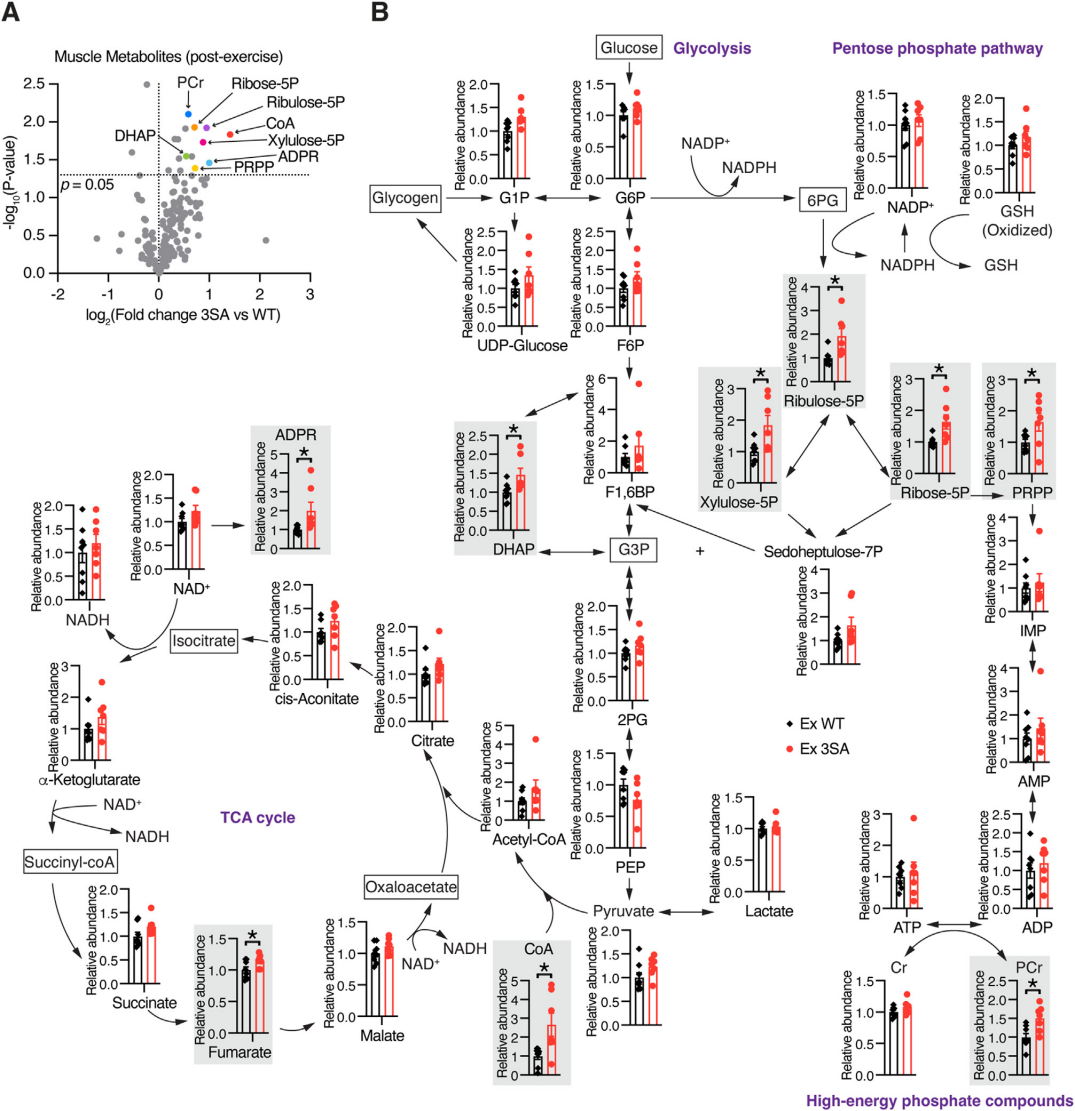
**ERRα drives alternative metabolic paths in exercising muscle.** (A) Volcano plot of exercised skeletal muscle metabolome of ERRα^3SA^ mice versus WT mice, n = 7–8. DHAP: Dihydroxyacetone phosphate; PCr: Phosphocreatine; ADPR: Adenosine-5′-diphosphate ribose; PRPP: Phosphoribosyl pyrophosphate; CoA: Coenzyme A. (B) Relative levels of metabolic intermediates mapped onto the pathways associated with glycolysis, TCA cycle, pentose phosphate pathway and high-energy phosphate compounds in skeletal muscle of exercised ERRα^3SA^ and WT littermates, n = 7–8. Significantly altered metabolites were highlighted by gray squares. ADP: Adenosine-5′-diphosphate; AMP: Adenosine-5′-monophosphate; ATP: Adenosine-5′-triphosphate; Cr: Creatine; G1P: Glucose-1-phosphate; G3P: Glucose-3-phosphate; G1P: Glucose-6-phosphate; GSH: Glutathione; F6P: Fructose-6-phosphate; F1,6BP: Fructose 1,6-bisphosphate; IMP: Inosine-5′-monophosphate; NAD^+^: Nicotinamide adenine dinucleotide; NADH: Nicotinamide adenine dinucleotide hydride; NADP: Nicotinamide adenine dinucleotide phosphate; NADPH: Nicotinamide adenine dinucleotide hydride phosphate; PCr: Phosphocreatine; 2 PG: 2-Phosphoglyceric acid; PEP: Phosphoenolpyruvic acid. Data in B are presented as means ± SEM (B), ∗p < 0.05, unpaired two-tailed Student's t test.

Given that ERRα 3SA mutation promotes metabolic reprogramming that accommodates energy demand for endurance exercise, we next used liquid chromatography-mass spectrometry (LC-MS) to profile muscle metabolites of exercised ERRα^3SA^ mice and their littermate controls. As anticipated, intermediates of glycolysis showed no obvious differences between genotypes upon exercise, except for DHAP (Figure 6A,B), which is known to be associated with enhanced endurance exercise capacity [51] and was evidently increased in ERRα^3SA^ muscle. Interestingly, exercised ERRα^3SA^ mice demonstrated concurrently upregulated intermediates of the pentose phosphate pathway, another glucose metabolic pathway, including Ribulose-5P (1.93 FC), Ribose-5P (1.64 FC), Xylulose-5P (1.84 FC), and nucleotide synthesis precursor PRPP (1.65 FC) (Figure 6A,B). Of note, coenzyme A (CoA), required for skeletal muscle FA activation and acetyl-CoA production, was significantly upregulated in exercised ERRα^3SA^ mice, in line with elevated expression of the gene encoding the rate-determining CoA biosynthesis enzyme, *Pank1*, as well as increased occupancy of ERRα^3SA^ protein to this gene locus (Figure 6A,B; Suppl. Fig. 6A,B). TCA cycle intermediates tended to be elevated but did not reach statistical significance, except fumarate (Figure 6B). NADH, a reducing agent that couples TCA cycle with OxPhos, showed no significant difference between genotypes, neither did NAD^+^ level or NAD^+^/NADH ratio (Figure 6B; Suppl. Fig. 6C), which are usually elevated by enhanced mitochondrial OxPhos. This discrepancy likely resulted from increased NAD^+^ hydrolysis for the synthesis of Adenosine diphosphate ribose (ADPR) (Figure 6A,B), an adenine-containing nucleotide known to regulate cellular Ca^2+^ homoeostasis [52]. Likewise, although exercised ERRα^3SA^ mice showed similar muscle ATP contents and ATP/ADP ratio compared with their littermate controls, ERRα^3SA^ mice possessed significantly upregulated high-energy phosphate compound phosphocreatine (PCr), seemingly derived from accelerated reformation fueled by aerobic ATP production (Figure 6A,B; Suppl. Fig. 6D). Notably, skeletal muscle PCr recovery from exercise has been reported to be a reliable index of mitochondrial oxidative capacity [53].

## 4 Discussion

Muscle response to physical exercise requires the integration of diverse signaling events, making it intriguing to decipher a class of master regulators central to the exercising network. Knockout studies have established a critical role of ERRα in controlling muscle exercise transcriptome [14], however the molecular mechanisms underlying the positive actions of ERRα on exercise performance remained elusive. Here, we harnessed to use of a mouse model with genetic disruption of three ERRα phosphosites (3SA) causing increased ERRα protein stability, an effect also observed by the stimulatory actions of exercise, to study the benefits of ERRα hyperactivation on exercise capacity.

ERRα^3SA^ mutation uncovered a large set of previously unknown ERRα targets exclusively regulated upon ERRα dephosphorylation, together with a reprogrammed muscle transcriptome that partially mimics the impacts of exercise. These molecular events together trigger skeletal muscle aerobic transformation represented by increased oxidative myofibers, angiogenesis, enhanced mitochondrial biogenesis and turnover, higher lactate and fat oxidation, increased OxPhos and accelerated PCr resynthesis, as well as activation of the pentose phosphate pathway, eventually augmenting endurance capacity (Suppl. Fig. 7). The ERRα^3SA^ mice also display enhanced motor coordination ability as measured by steady-accelerating and constant-speed rotarod tests. This observation suggests that increased ERRα^3SA^ activity outside of skeletal muscle, such as in the neuronal system, may also contribute to the observed phenotype. Indeed, ERRα-null mice exhibit schizophrenia-like behaviors and has been shown to be necessary for PGC-1α-dependent gene expression in the brain [54].

The observed profound impacts of dephosphorylation on ERRα chromatin occupancy and target gene expression, undoubtedly largely stemming from stabilized ERRα protein, may involve other mechanisms requiring further study such as differential recruitment of co-regulators that contribute to these genomic alterations. For instance, PGC-1α, a potent protein ligand for ERRα [55,56], has emerged as a central regulator orchestrating many of the exercise responses. It is well-known that ERRα and PGC-1α simultaneously act on a subset of genes predominantly regulating mitochondrial biogenesis, lipid oxidation, TCA cycle, and OxPhos [19]. ERRα^3SA^ mutation amplifies exercise-induced elevation of muscle PGC-1α, indicating that ERRα is another potent factor in a functional feedforward loop controlling PGC-1α expression in muscle [57], which synergistically modulates muscular responses during exercise. Furthermore, genetic phospho-attenuation of ERRα might favor its exchange of co-repressors for co-activators. For instance, muscle-specific loss of the classic ERRα corepressor NCoR1 enhances mitochondrial oxidative metabolism and exercise endurance, accompanied by selective ERRα activation [18].

Increases in oxidative slow-twitch myofibers could prevent muscle wasting [58], implying the potential of targeting fiber-type regulators for the treatment of diseases related to muscle wasting and frailty. The intracellular signals specifying muscle fiber types have only been partially elucidated. Surprisingly, while ERRα knockout mice display a decrease in muscle mass [14], specific loss of ERRα has minimal effects on muscle fiber types [13], likely compensated by other redundant transcription factors, including the ERRγ isoform [59]. Indeed, combined loss of ERRα and ERRγ leads to more pronounced adverse effects on muscle functions [24,25]. By contrast, ERRα phospho-mutation at birth activates genetic programs characteristic of oxidative slow-twitch muscle fibers, highlighting ERRα^3SA^ mice as a valuable model to unmask undiscovered ERRα functions. While transgenic overexpression of ERRγ in skeletal muscle is found accompanied by an upregulation of ERRβ but not ERRα mRNA expression [59], enhanced ERRα stabilization observed in 3SA muscle has no significant impact on the transcription of other ERR isoforms (Suppl. Fig. 8A,B).

Well-trained muscle displays significantly improved muscle oxidative capacity and is more flexible in switching between different nutrient sources. In addition, upon exercise, muscle capably absorbs and metabolizes circulating fuel substrates either derived from diet or released from peripheral tissues [60]. Impaired lactate and fat metabolism are critical contributors to the development of metabolic disorders. Lactate has long been considered as a waste side product of high-intensity exercise that exacerbates muscle fatigue and limits exercise performance [43]. Interestingly, lactate possesses many crucial functions that were under-appreciated, including restricting mitochondrial fatty acid uptake and oxidation, allosterically suppressing adipose lipolysis, as well as mediating tissue-to-tissue communications [43,61]. Our study supports lactate as a critical marker of exercise tolerance and key signaling molecule. We demonstrate that ERRα hyperactivation enhances lactate utilization via its oxidation to pyruvate through upregulation of Ldh1 isoenzyme activity. Our findings reinforce previous reports implicating ERRα in the direct control of Ldh complex remodeling in muscle and its positive regulation of lactate oxidation in breast cancer cells [62,63]. The higher lactate clearance capacity of ERRα^3SA^ mice is accompanied by boosted FAO and elevated peripheral fat mobilization into muscle, highlighting the potent role of ERRα in maintaining a dynamic equilibrium between lactate and fat metabolism to achieve systemic homeostasis.

Obese and type 2 diabetes patients display “metabolic inflexibility” manifested by impaired insulin-stimulated skeletal muscle glucose oxidation [64,65]. ERRα was indicated to inhibit skeletal muscle glucose catabolism by transcriptionally activating Pdk4, a negative regulator of glucose oxidation that is elevated in diabetes [66,67]. Herein we evidenced increased Pdk4 levels in ERRα^3SA^ muscle together with elevated ERRα binding to the *Pdk4* promoter, implying a regulatory role of ERRα in metabolic flexibility. On the other hand, *Pdk4* is a well-characterized gene induced by exercise training in skeletal muscle to enhance exercise endurance via preserving glucose [14,68]. Therefore, elevated muscle Pdk4 levels might simultaneously contribute to defects in postprandial glucose disposal and increase endurance performance. Further, we previously observed an elevated RER in HFD-fed ERRα^3SA^ mice along with upregulated hepatic glycolysis and lipogenesis [28], implying their defects in using fat as an energy fuel. Similarly, but via a different mechanism, ERRα KO mice displayed a higher RER, due to insufficient oxidation of glucose and fat secondary to impaired mitochondrial function, consequently resulting in elevated energy demand from glycolysis [14]. It is also well-known that muscle contraction increases insulin sensitivity and glucose uptake, with the imported glucose primarily being used for glycogen re-storage [69,70]. Exercise indeed alleviated the impaired glucose metabolism of ERRα^3SA^ mice, independent of effects on muscle glycogen content. Instead, exercised ERRα^3SA^ mice showed evident muscle activation of the pentose phosphate pathway, an alternative route metabolising glucose. Activation of the pentose phosphate pathway has been reported to participate in the resistance against reactive oxygen species (ROS) and production of substrates for nucleic acid and lipid biosynthesis [71]. Our findings of ERRα-dependent regulation of the pentose phosphate pathway provide new insights into physiological muscle adaptation to exercise and corroborate previous discovery of ERRα as a ROS sensor [72].

Collectively, our study demonstrates an essential role of the ERRα phosphorylation state in the control of muscle functions and energy metabolism during endurance exercise, highlighting ERRα hyperactivation as a potential therapeutic target to improve exercise performance and achieve long-term exercise benefits [73]. Future work will help delineate the exact contribution of post-translational mechanisms contributing to exercise-induced ERRα stabilization and overall activity. Although this study has a focus on muscle, adaptive exercise response involves whole-body biology, including the nervous system. Thus, it will be important to dissect tissue-specific ERRα phosphorylation/dephosphorylation signaling pathways for more precise targeting of ERRα function in metabolic control.

## 5 Conclusions

We have demonstrated that increased ERRα activity via protein stabilization observed in phospho-deficient ERRα^3SA^ mice enhances exercise capacity upheld by a favorable oxidative metabolic profile manifested by increased oxidative muscle fiber types, angiogenesis, mitochondrial biogenesis, FAO, and lactate clearance via oxidation to pyruvate.

## Author contributions

**Hui Xia**: Conceptualization, Investigation, Formal analysis, Writing - Original draft; **Charlotte Scholtes**: Investigation, Formal analysis, Writing - Review and editing; **Catherine R. Dufour**: Investigation, Formal analysis, Data curation, Writing - Review and editing; **Christina Guluzian**: Investigation; **Vincent Giguère**: Conceptualization, Writing - Review and editing, Supervision, Funding acquisition.

## Declaration of Competing Interest

The authors declare that they have no known competing financial interests or personal relationships that could have appeared to influence the work reported in this paper.

## Data Availability

Data will be made available on request.
