# Supplementary material for: ERRα fosters running endurance by driving myofiber aerobic transformation and fuel efficiency
Source: Mol Metab. 2023 Oct 5;78:101814. doi: 10.1016/j.molmet.2023.101814 (PMC10590867; doi:10.1016/j.molmet.2023.101814)
Supplement: Multimedia component 4 [file mmc4.pdf]

ERRα fosters running endurance by driving myofiber aerobic transformation and fuel efficiency

Hui Xia, Charlotte Scholtes, Catherine R. Dufour, Christina Guluzian and Vincent Giguère

Supplemental Materials

Supplemental Figures 1-8

Supplemental Tables 1-3 (Excel Files)

Supplemental Figure 1

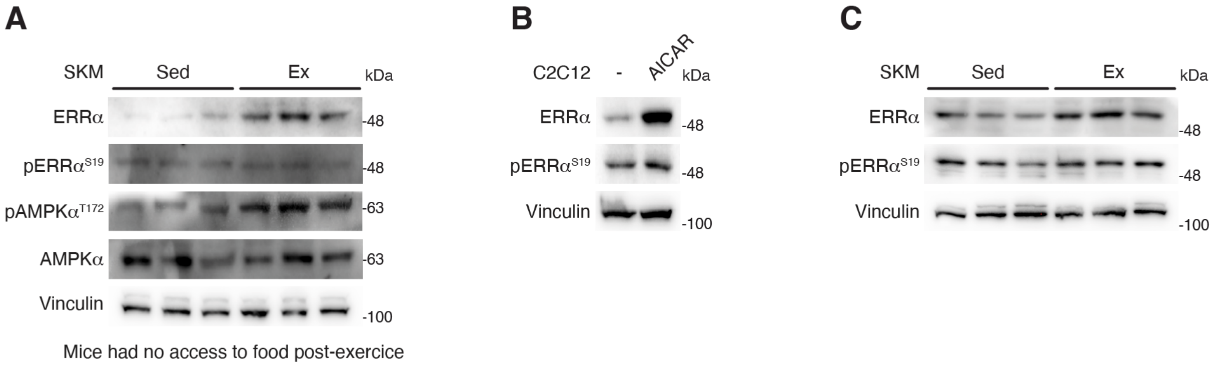

Supplemental Figure 1: (A) Immunoblots of indicated proteins in SKM of WT and ERRα^{3SA} mice post the treadmill exhaustion test. Mice had only access to water, but no access to food during the recovery period post exercise to eliminate any potential post-feeding insulin-mediated induction of ERRα. Each lane represents one mouse, n = 3. (B) Immunoblots of ERRα and pERRα^{S19} proteins in C2C12 muscle cells treated with 1 mM AICAR for 6 h. (C) Immunoblots of ERRα and pERRα^{S19} proteins in SKM of WT and ERRα^{3SA} mice post the treadmill exhaustion test. Each lane represents one mouse, n = 3.

Supplemental Figure 2

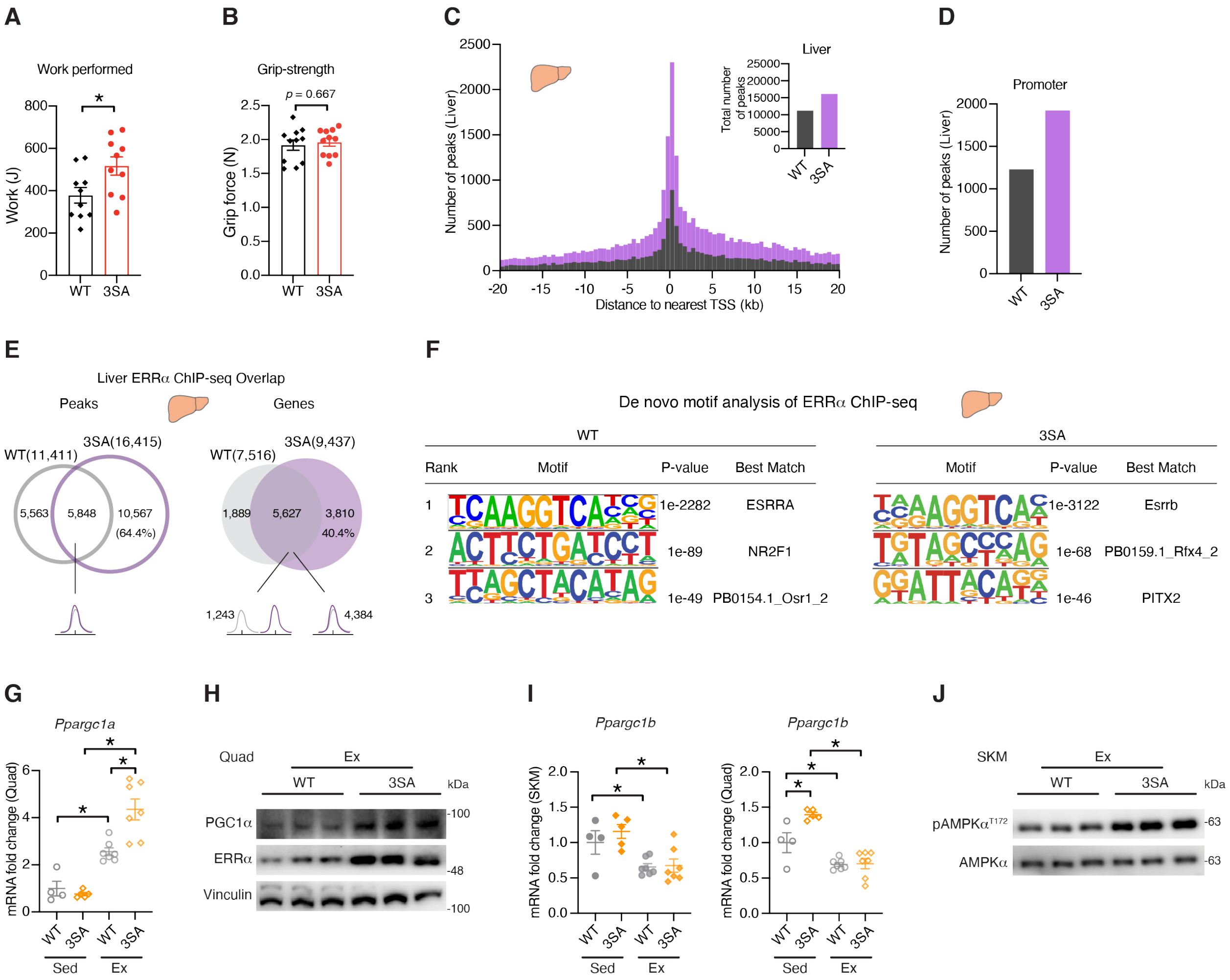

Supplemental Figure 2: (A) Work performed by WT and ERRα^{3SA} mice during the treadmill exhaustion test (n = 10). (B) Four-limbs peak force tested in ERRα^{3SA} and their WT littermates (n = 11) using a grip strength meter. (C) Distribution of liver ERRα ChIP-seq peaks ± 20 kb relative to the transcription start site (TSS) of the nearest gene identified in ERRα^{3SA} and WT littermates. Inner histogram shows the total number of peaks bound by ERRα within ±20 kb of TSS. (D) Number of promoter-annotated liver ERRα ChIP-seq peaks. (E) Overlap of ERRα ChIP-seq binding peaks (left) and target genes (right) within ±20 kb of TSS identified in the liver of ERRα^{3SA}

and WT littermates. (F) De novo motif computational discovery of ERRα-bound sequences (within ±20 kb of TSS) in the liver of ERRα^{3SA} and WT littermates. (G) *Ppargc1a* mRNA levels in quadriceps of WT and ERRα^{3SA} mice in the sedentary state or post the treadmill exhaustion test, n = 4-7. (H) Immunoblots of PGC-1α proteins in quadriceps of WT and ERRα^{3SA} mice post the treadmill exhaustion test. Each lane represents one mouse, n = 3. (I) *Ppargc1b* mRNA levels in both oxidative skeletal muscle and quadriceps of WT and ERRα^{3SA} mice in the sedentary state or post the treadmill exhaustion test, n = 4-7. (J) Phosphorylated (T172) and total AMPKα protein levels in skeletal muscle from WT and ERRα^{3SA} mice post the treadmill exhaustion test. Each lane represents one mouse, n = 3. Data are presented as means ± SEM, *$p < 0.05$, unpaired two-tailed Student's t test (A,B,G,I).

Supplemental Figure 3

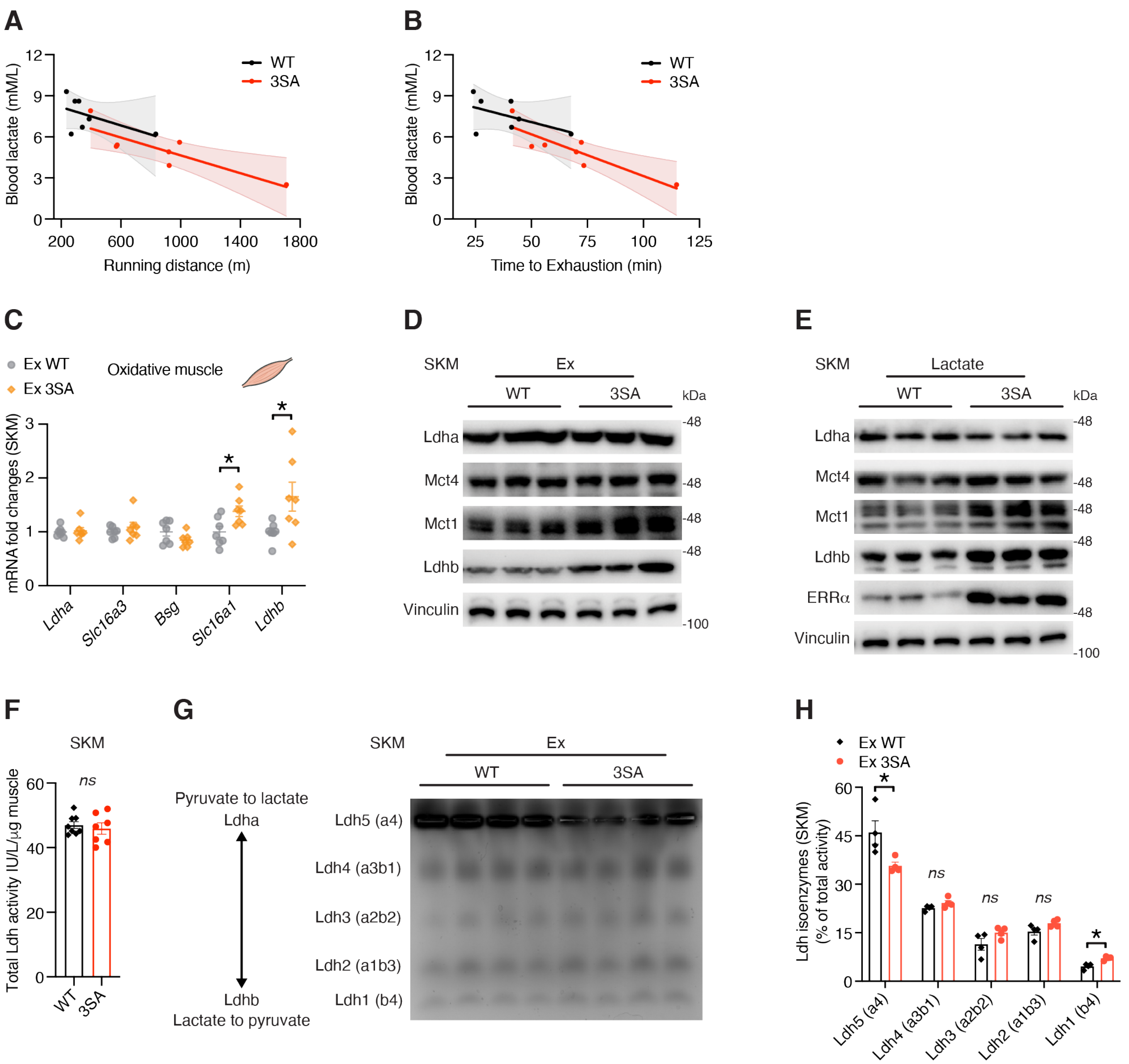

Supplemental Figure 3: (A,B) Regression plots of blood lactate vs. either running distance (A) or time to exhaustion (B) in WT and ERRα^{3SA} mice post the treadmill exhaustion test (n=7). (C,D) Skeletal muscle mRNA (C, n = 7) and protein (D, each lane represents one mouse, n = 3) levels of genes involved in lactate metabolism of WT and ERRα^{3SA} mice post the treadmill exhaustion test. (E) Skeletal muscle protein levels of genes involved in lactate metabolism of WT and ERRα^{3SA}

mice post lactate stimulation. Each lane represents one mouse, n = 3. (F) Total Ldh activity in skeletal muscle of WT and ERRα^{3SA} mice post the treadmill exhaustion test (n=7-8). (G) Non-denaturing agarose gel electrophoresis determination of Ldh isoenzyme composition in skeletal muscle of WT and ERRα^{3SA} mice post the treadmill exhaustion test (each lane represents one mouse, n = 4). (H) Quantification of individual Ldh isoenzymes identified in (G) expressed as percent of total Ldh activity. Data are presented as means ± SEM, *$p < 0.05$, unpaired two-tailed Student's t test (C,F,H). ns: not significant.

Supplemental Figure 4

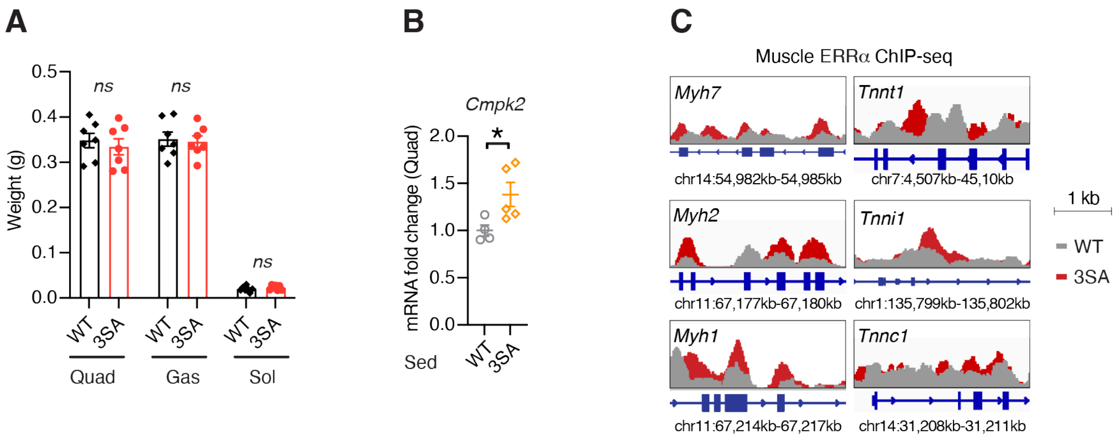

Supplemental Figure 4: (A) Weights of distinct muscles of ERRα^{3SA} mice and WT littermates, n = 7. Quad: quadriceps; Gas: gastrocnemius; Sol: soleus. (B) *Cmpk2* mRNA levels in quadriceps of ERRα^{3SA} and WT littermates, n = 4-5. (C) WT and ERRα^{3SA} muscle ChIP-seq tracks at the indicated genes. Data are presented as means ± SEM, *$p < 0.05$, unpaired two-tailed Student's t test (A,B). ns: not significant.

Supplemental Figure 5

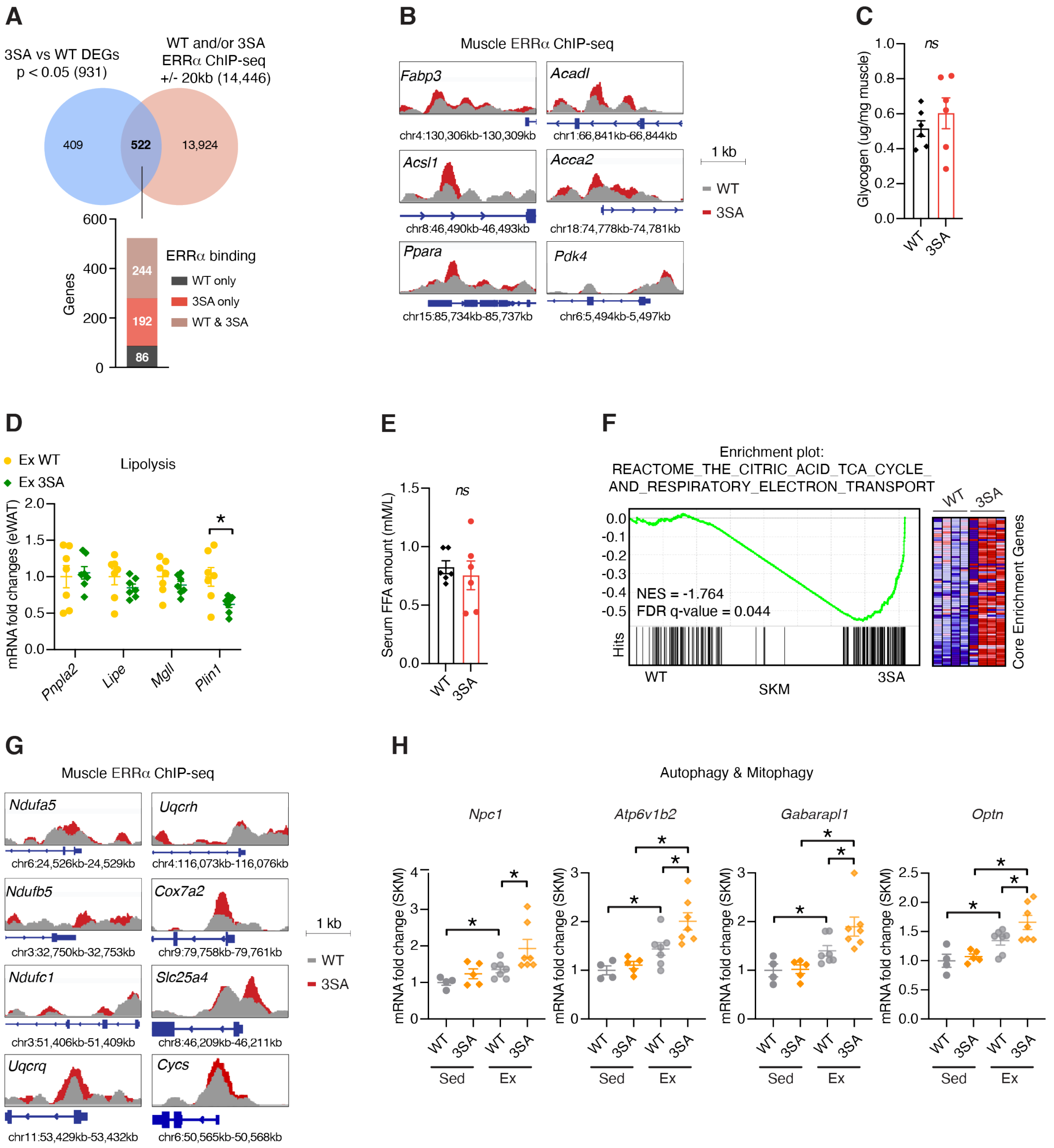

Supplemental Figure 5: (A) Venn diagram and bar chart showing that 56% of skeletal muscle ERRα^{3SA} DEGs ($p < 0.05$) harbor an ERRα binding site ± 20kb of gene TSSs as identified by

ChIP-seq in either WT or ERRα^{3SA} mice. (B) WT and ERRα^{3SA} muscle ChIP-seq tracks at the indicated genes. (C) Muscle glycogen contents of WT and ERRα^{3SA} mice post the treadmill exhaustion test, n = 6. (D) Relative mRNA levels of lipolysis genes in epididymal white adipose tissue (eWAT) of exercised ERRα^{3SA} and WT littermate controls, n = 7. (E) Serum free fatty acid (FFA) contents of WT and ERRα^{3SA} mice post the treadmill exhaustion test, n = 6. (F) GSEA showing an upregulated Reactome gene signature from the Molecular Signature Database (MSigDB, mouse version 2023.1) related to the TCA cycle and electron transport chain in ERRα^{3SA} vs WT muscle transcriptomes. (G) WT and ERRα^{3SA} muscle ChIP-seq tracks at the indicated genes. (H) Relative mRNA levels of genes related to autophagy and mitophagy in skeletal muscle of WT and ERRα^{3SA} mice in the sedentary state or post the treadmill exhaustion test, n = 4-7. Data are presented as means ± SEM, $*p < 0.05$, unpaired two-tailed Student's t test (C-E,H). ns: not significant.

Supplemental Figure 6

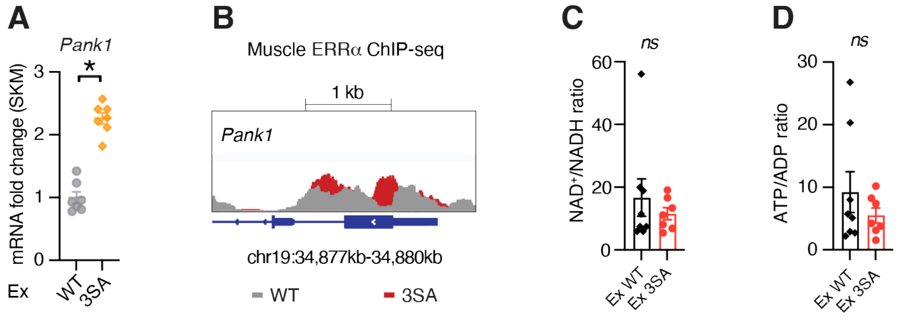

Supplemental Figure 6: (A) Relative *Pank1* mRNA levels in skeletal muscle of WT and ERRα^{3SA} mice post the treadmill exhaustion test, n = 7. (B) WT and ERRα^{3SA} muscle ChIP-seq tracks at *Pank1*. (C,D) NAD^+/NADH (C) and ATP/ADP (D) ratio in skeletal muscle of exercised ERRα^{3SA} and WT littermates, n = 7-8. Data are presented as means ± SEM, $^*p < 0.05$, unpaired two-tailed Student's t test (A,C,D). ns: not significant.

Supplemental Figure 7

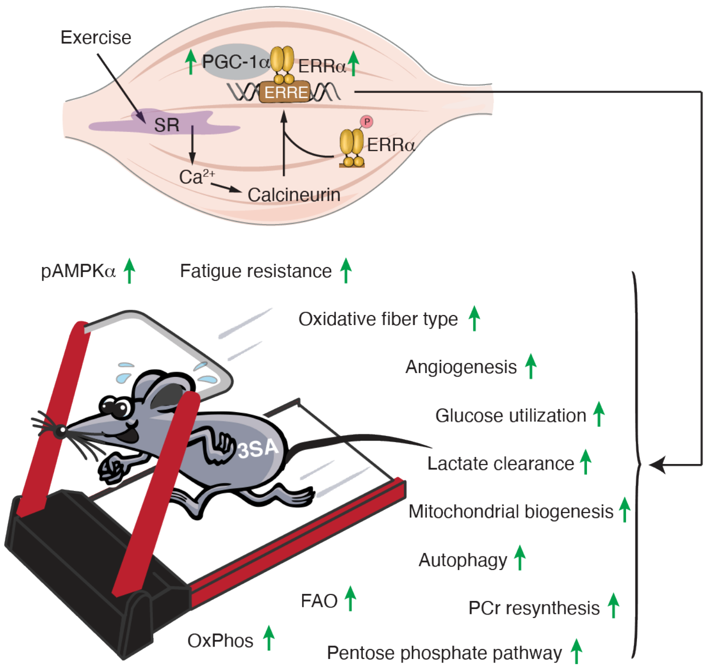

Supplemental Figure 7: ERRα activation drives metabolic conditioning of skeletal muscle to endurance exercise. Schematic summary of the physiological adaptations resulting from hyperactivation of ERRα activity observed in ERRα^{3SA} mice (left) and changes in ERRα-dependent genes involved in signaling pathways regulating muscle function and metabolic homeostasis during exercise (right).

Supplemental Figure 8

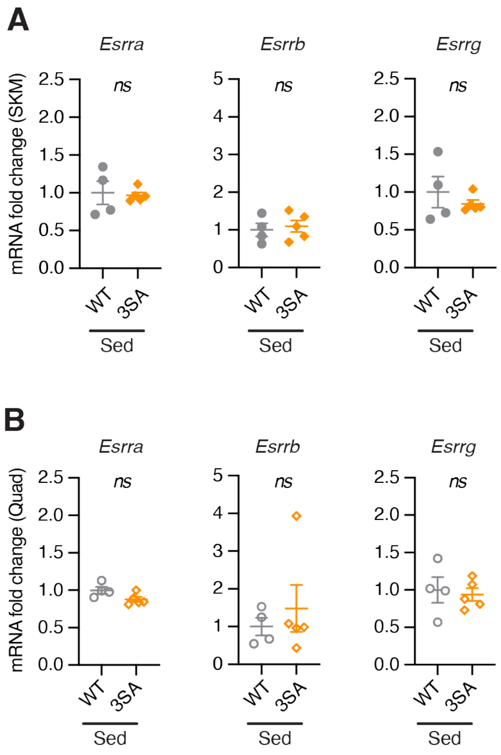

Supplemental Figure 8: ERR isoform transcript profiles in muscle of WT and ERRα^{3SA} mice. (A,B) Relative mRNA levels encoding ERR isoforms ERRα, ERRβ, and ERRγ in skeletal muscle (A) and quadriceps (B) of WT and ERRα^{3SA} mice in the sedentary state or post the treadmill exhaustion test, n = 4-7. Data are presented as means ± SEM, $*p < 0.05$, unpaired two-tailed Student’s t test (A,B). ns: not significant.

Supplemental Table 1: ERRα ChIP-seq in WT and ERRα^{3SA} mouse skeletal muscle.

Supplemental Table 2: ERRα ChIP-seq in WT and ERRα^{3SA} mouse liver.

Supplemental Table 3: Mouse RT-qPCR primer sets used in this study.
